# Itm2a expression marks periosteal skeletal stem cells that contribute to bone fracture healing

**DOI:** 10.1172/JCI176528

**Published:** 2024-09-03

**Authors:** Wenhui Xing, Heng Feng, Bo Jiang, Bo Gao, Jiping Liu, Zaiqi Xie, Yazhuo Zhang, Xuye Hu, Jun Sun, Matthew B. Greenblatt, Bo O. Zhou, Weiguo Zou

**Affiliations:** 1 Key Laboratory of RNA Innovation, Science and Engineering, CAS Center for Excellence in Molecular Cell Science, Shanghai Institute of Biochemistry and Cell Biology, University of Chinese Academy of Sciences, Chinese Academy of Sciences, Shanghai, China.; 2 Hainan Academy of Medical Sciences, Hainan Medical University, Hainan, China.; 3 Institute of Orthopaedic Surgery, Xijing Hospital, Air Force Military Medical University, Xi’an, Shaanxi, China.; 4 Stem Cell Translational Research Center, Tongji Hospital, School of Medicine, Tongji University, Shanghai, China.; 5 Department of Pathology and Laboratory Medicine, Weill Cornell Medicine, New York, New York, USA.; 6 Research Division, Hospital for Special Surgery, New York, New York, USA.; 7 Key Laboratory of Multi-Cell Systems, CAS Center for Excellence in Molecular Cell Science, Shanghai Institute of Biochemistry and Cell Biology, University of Chinese Academy of Sciences, Chinese Academy of Sciences, Shanghai, China.; 8 State Key Laboratory of Experimental Hematology, Institute of Hematology and Blood Diseases Hospital, Chinese Academy of Medical Sciences, Tianjin, China.; 9 Institute of Microsurgery on Extremities, and Department of Orthopedic Surgery, Shanghai Sixth People’s Hospital Affiliated to Shanghai Jiao Tong University School of Medicine, Shanghai, China.

**Keywords:** Bone biology, Bone disease

## Abstract

The periosteum contains skeletal stem/progenitor cells that contribute to bone fracture healing. However, the in vivo identity of periosteal skeletal stem cells (P-SSCs) remains unclear, and membrane protein markers of P-SSCs that facilitate tissue engineering are needed. Here, we identified integral membrane protein 2A (*Itm2a*) enriched in SSCs using single-cell transcriptomics. Itm2a^+^ P-SSCs displayed clonal multipotency and self-renewal and sat at the apex of their differentiation hierarchy. Lineage-tracing experiments showed that *Itm2a* selectively labeled the periosteum and that Itm2a^+^ cells were preferentially located in the outer fibrous layer of the periosteum. The Itm2a^+^ cells rarely expressed *CD34* or *Osx*, but expressed periosteal markers such as *Ctsk*, *CD51*, *PDGFRA*, *Sca1*, and *Gli1*. Itm2a^+^ P-SSCs contributed to osteoblasts, chondrocytes, and marrow stromal cells upon injury. Genetic lineage tracing using dual recombinases showed that *Itm2a* and *Prrx1* lineage cells generated spatially separated subsets of chondrocytes and osteoblasts during fracture healing. Bone morphogenetic protein 2 (*Bmp2*) deficiency or ablation of Itm2a^+^ P-SSCs resulted in defects in fracture healing. ITM2A^+^ P-SSCs were also present in the human periosteum. Thus, our study identified a membrane protein marker that labels P-SSCs, providing an attractive target for drug and cellular therapy for skeletal disorders.

## Abstract 2

Single-cell transcriptomics identifies membrane protein ITM2A as a marker that labels periosteum skeletal stem cells.

## Introduction

Skeletal stem cells (SSCs), as established by Paolo Bianco and colleagues, have the ability to generate colony-forming units and to differentiate into bone, cartilage, adipocyte,s and stromal cells (1, 2). SSCs are identified by the cell-surface markers CD45^–^TER119^–^Tie2^–^Thy^–^6C3^–^CD105^–^αV^+^CD200^+^ in mice and Lin^–^PDPN^+^CD146^–^CD73^+^CD164^+^ in humans (3, 4). The stem cells involved in bone fracture repair are mainly derived from the periosteum, bone marrow, and endosteum (5). Bone graft experiments supported the hypothesis that the periosteum is a major stem cell source and is critical for bone fracture healing (6, 7). Genetic mouse models and lineage-tracing techniques have shown that several Cre or Cre-ERT2 mice lines driven by different marker genes, such as *Prrx1* (6, 8), *Osx* (9), *Ctsk* (10), *Mx1*, *Acta2* (11), *Gli1* (12, 13), and *PDGFRA* (14, 15) could label periosteal cells during bone regeneration. It is believed that the cells labeled by these marker genes are in correlation with periosteal skeletal stem cells (P-SSCs), which could maintain and repair postnatal cortical bone. However, these different markers could label multiple other lineages of cells besides P-SSCs, which may be overlapped in part by the different markers. Another possibility is that different marker genes label different lineages of P-SSCs, which jointly contribute to intramembranous and endochondral ossification during bone fracture healing. It is critical to identify rare P-SSCs and establish ways to harvest these cells for cellular therapy for skeletal disorders.

*Prrx1* is a key transcription factor expressed during early limb development (16). *Prrx1-Cre* labels osteochondral progenitor cells in the developing limb buds and in a subpopulation of periosteal cells (16). It has been shown that the *Prrx1* lineage is a major cellular source for bone fracture healing (6, 8, 17, 18) and that ablation of Prrx1^+^ cells affects bone fracture healing (8). The above studies have demonstrated that *Prrx1-Cre* labels a group of SSCs that are critical for fracture healing, but how to isolate and study this group of cells remains unclear.

Given their capacity for self-renewal and differentiation into various mature cell types, stem cells are considered essential for tissue regeneration in musculoskeletal conditions (19). The identification of membrane protein markers becomes imperative for enabling efficient P-SSC cell sorting in downstream tissue engineering applications. To identify the bona fide SSCs in *Prrx1* lineage periosteal cells, we separated Ai9^+^ periosteal cells from 4-week-old *Prrx1-Cre R26-Ai9* mice and performed single-cell RNA-Seq (scRNA-Seq). Combined with the analysis of the SSC subpopulation, an integral membrane protein 2A (*Itm2a*) was identified as a key marker enriching SSCs. ITM2A is a 263 amino acid protein with 1 transmembrane domain (20). *Itm2a* was isolated by a complementary DNA (cDNA) library subtraction of in vitro cultivated murine mandibular condyles and found to be expressed in osteogenic tissues (21). However, the role of Itm2a in periosteum has not been determined.

In this study, we developed an inducible Cre-knockin transgenic line at the *Itm2a* locus, allowing for the labeling of P-SSCs. In vivo and in vitro experiments supported the hypothesis that *Itm2a*-expressing cells serve as P-SSCs contributing to bone fracture healing. We also found that ITM2A could enrich a high percentage of SSCs in human periosteum samples. The identification of P-SSCs labeled by the membrane protein ITM2A indicates the therapeutic potential of ITM2A^+^ cells in bone fracture treatment.

## Results

### Itm2a enriches SSCs in Prrx1 lineage periosteal cells from scRNA-Seq analysis.

**Figure 1:**
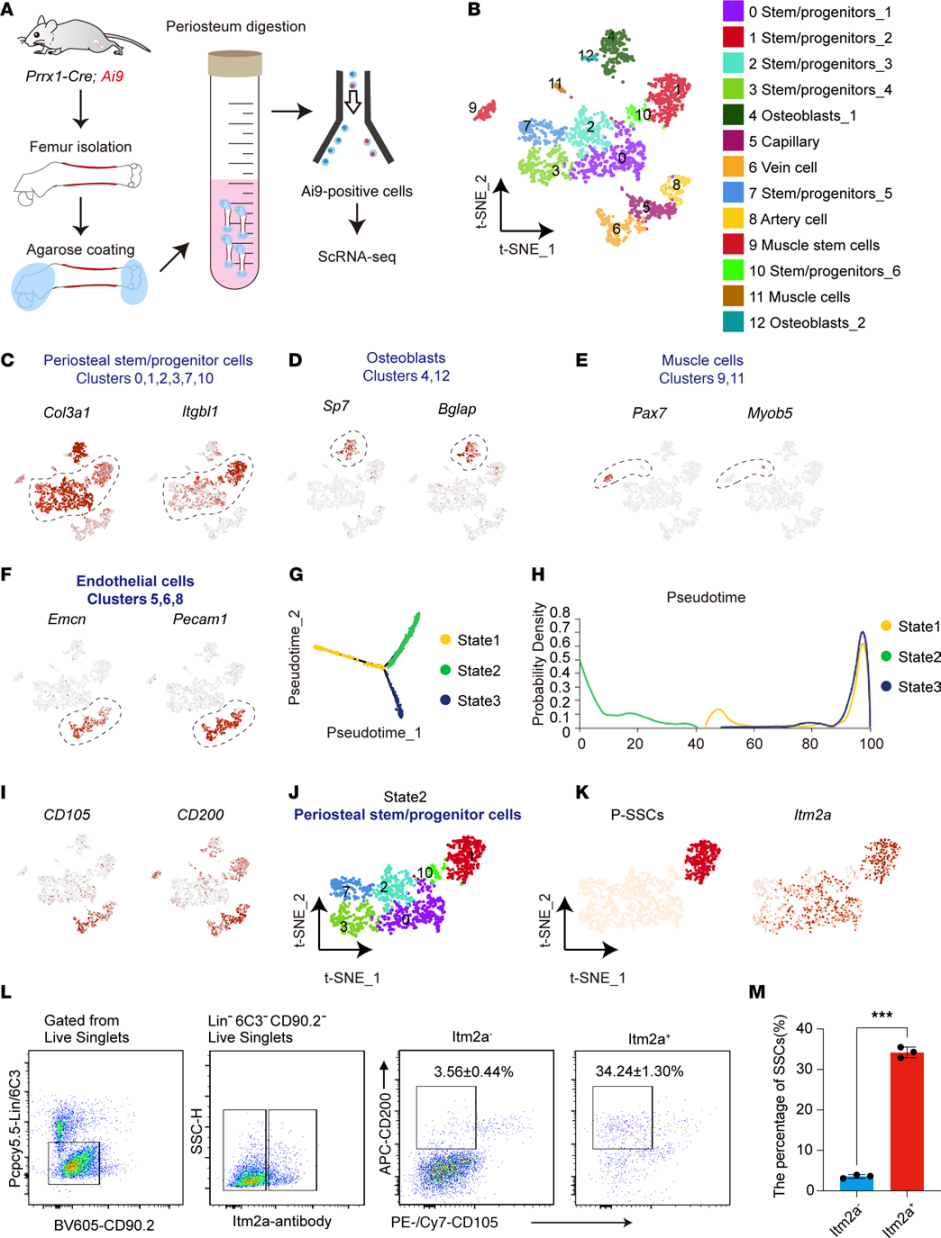
scRNA-Seq analysis shows that Itm2a can enrich SSCs in *Prrx1* lineage periosteal cells. (**A**) The isolation and scRNA-Seq workflow of Ai9^+^ periosteal cells from *Prrx1-Cre R26-Ai9* mice. (**B**) *t*-SNE plot of collected Ai9^+^ cells. Cells were clustered into 13 subpopulations: stem/progenitor cells (clusters 0–3, 7, and 10), osteoblasts (clusters 4 and 12), ECs (clusters 5, 6, and 8), and muscle cells (clusters 9 and 11). (**C**–**F**) Feature plots showing the representative marker distribution in different cell types: periosteal stem/progenitor cells (*Col3a1* and *Itgbl1*) (**C**), osteoblasts (*Sp7* and *Bglap*) (**D**), muscle cells (*Pax7* and *Myob5*) (**E**), and ECs (*Emcn* and *Pecam1*) (**F**). (**G** and **H**) Pseudotime analysis of the 3 states of Ai9^+^ cells. (**I**) Feature plots of *CD105* and *CD200* expression in Ai9^+^ cells. (**J**) *t*-SNE plot of the periosteal stem/progenitor cells (state 2, clusters 0, 1, 2, 3, 7, and 10). (**K**) Feature plots showing the distribution of *Itm2a* in P-SSCs. (**L**) Flow cytometric analysis of SSCs and progenitors in the murine periosteum. (**M**) Percentage of SSCs in Itm2a^+^ and Itm2a^–^ cell populations. Data are presented as the mean ± SD. *n* = 3. ***P < 0.001, by unpaired *t* test.

To define the bona fide SSCs in *Prrx1* lineage periosteal cells, we performed scRNA-Seq on Ai9^+^ periosteal cells from 4-week-old *Prrx1-Cre*; *R26-Ai9* mice. Ai9 reporter–positive cells were sorted from the femoral shaft periosteum of 4-week-old male mice using flow cytometry after agarose coating, collagenase digestion, and erythrocyte lysis, followed by scRNA-Seq analysis on the 10X Genomics platform (Figure 1A). We noticed that *Prrx1-Cre* also labeled a small number of CD45^+^ cells, which is consistent with a previous study (22). After quality control, we obtained 2,169 CD45^–^ single cells from Ai9^+^ cells.

After batch effect correction, we performed an integrated analysis of *Prrx1-Cre* lineage cells, which revealed 13 subsets using nonlinear dimensionality reduction displayed with *t*-distributed stochastic neighbor embedding (*t*-SNE) (Figure 1B). We detected periosteal stem/progenitor cells (clusters 0, 1, 2, 3, 7, and 10 expressing *Col3a1* and *Itgbl1*, Figure 1C) (22). In addition to periosteal stem/progenitor cells, we identified subsets that included osteoblasts (clusters 4 and 12, expressing *Sp7* and *Bglap*), muscle cells (clusters 9 and 11, expressing *Pax7* and *Myob5*), and endothelial cells (ECs) (clusters 5, 6 and 8, expressing *Emcn* and *Pecam1*) (Figure 1, D–F). We next explored the heterogeneity of *Prrx1-Cre*–traced cells and found 3 states, and the pseudotime analysis of the 3 states showed a trajectory from state 2 (clusters 0, 1, 2, 3, 7, and 10) to state 1 and state 3 (Figure 1, G and H, and Supplemental Figure 1A; supplemental material available online with this article; https://doi.org/10.1172/JCI176528DS1). The *t*-SNE plot shows the distribution of SSC markers (*CD105* and *CD200*) and other known stem/progenitor markers (Figure 1I and Supplemental Figure 1B) in the *Prrx1-Cre*–traced cells, which defined cluster 1 as expressing markers consistent with P-SSCs (Figure 1J).

We also analyzed the expression of characteristic P-SSC genes (Supplemental Figure 1C) and found that *Prrx1* was expressed at high levels in P-SSCs. Among the genes with the highest expression in P-SSCs, *Itm2a* was the most specific membrane protein (ranked 14th, Supplemental Figure 1C). The feature plots show high *Itm2a* expression in P-SSCs (CD45^–^CD31^–^Ter119^–^6C3^–^CD90.2^–^CD105^–^CD200^+^) (Figure 1K). Using the same periosteum isolation method as for scRNA-Seq, we analyzed the proportion of SSCs in the murine periosteum and found that *Itm2a* could greatly enrich for periosteal SSCs (34.24% ± 1.3%) (Figure 1, L and M).

### Functional characterization of Itm2a^+^ P-SSCs.

**Figure 2:**
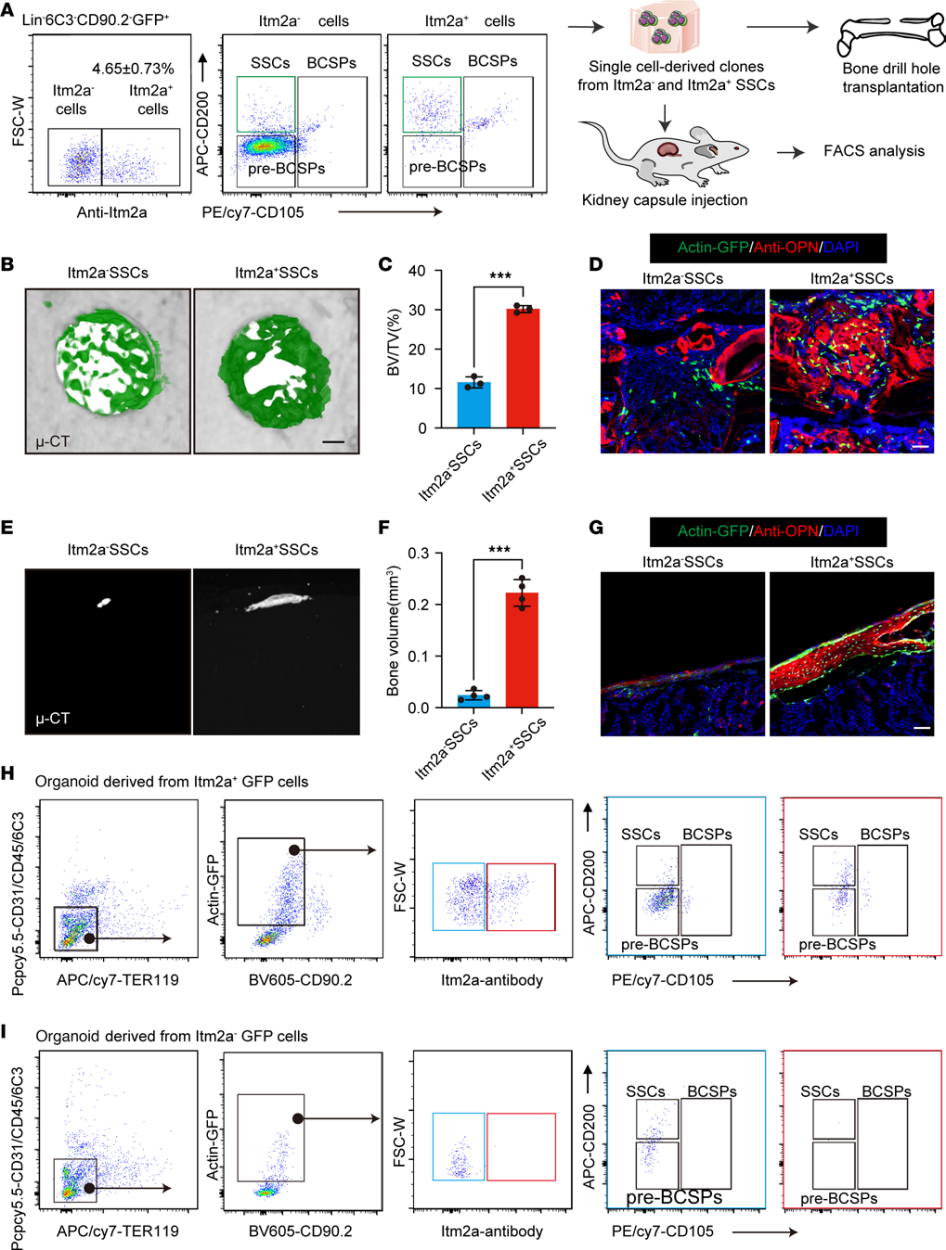
Functional characterization of Itm2a^+^ P-SSCs. (**A**) Workflow of the kidney capsule transplantation and bone drill-hole transplantation experiment using actin-GFP mice. Itm2a^–^ and Itm2a^+^ GFP^+^ SSCs were sorted, cultured, and transplanted in a kidney capsule injection assay and a bone drill-hole model. (**B**) Representative μ-CT images of long bone defects at day 7 after injury and transplantation. (**C**) Bone volume/total volume (BV/TV) quantification of injury sites were assessed for the quantification of bone healing. *n* = 4. ***P < 0.001, by unpaired, 2-tailed *t* test. (**D**) Representative immunostaining images of long bone defects 7 days after injury and transplantation. Scale bar: 50 μm. (**E**) Representative μ-CT images of the renal grafts 2 months after transplantation of Itm2a^–^ and Itm2a^+^ SSCs. (**F**) Bone volume (BV) quantification of the renal grafts from **G**. *n* = 3. ***P < 0.001, by 2-tailed, unpaired *t* test. (**G**) Representative immunostaining images of the renal grafts 2 months after transplantation of Itm2a^–^ and Itm2a^+^ SSCs. Scale bar: 50 μm. (**H** and **I**) FACS analysis of Itm2a^+^ (**H**) and Itm2a^–^ (**I**) SSC-derived cells after transplantation. FSC-W, forward scatter width. Data are presented as the mean ± SD.

To confirm that ITM2A is a membrane protein, we transfected 293 cells and found that ITM2A (green) colocalized with a cell membrane marker, Dil (red) (Supplemental Figure 2A). We performed FACS to study the properties of *Itm2a*-expressing periosteal cells. Four-week-old periosteal cells were harvested using the same method described above. Next, we performed a CFU-F experiment on Lin^–^Itm2a^–^ and Lin^–^Itm2a^+^ cells, and we observed a higher CFU-F ability in the Lin^–^Itm2a^+^ group (Supplemental Figure 2, B and C). Both Lin^–^Itm2a^–^ and Lin^–^Itm2a^+^ cells displayed in vitro clonal multipotency for chondrocytes, adipocytes, and osteoblasts (Supplemental Figure 2D). Flow cytometric analysis showed that *Itm2a* can greatly enrich immunophenotypic SSCs (Figure 1, L and M). Given this, we compared the stemness of Lin^–^6C3^–^CD90.2^–^CD105^–^CD200^+^ (hereafter referred to as Itm2a^+^ SSCs) and Lin^–^6C3^–^CD90.2^–^CD105^–^CD200^+^Itm2a^–^ (hereafter referred to as Itm2a^–^ SSCs) SSCs. The stem cell properties of the Itm2a^+^ SSCs were confirmed by renal subcapsular transplantation and bone drill hole transplantation (Figure 2A). Actin-GFP mice (which express GFP under the actin promoter) were used as a source of cells for transplantation into both the bone drill hole and renal subcapsular transplantation models. We sorted GFP-labeled Itm2a^–^ SSCs and Itm2a^+^ SSCs cells and then embedded these cells in Matrigel. The Matrigel-encapsulated cells were injected into cortical drill hole sites in the femoral diaphysis (Figure 2A). Micro-CT (μ-CT) analysis of mice 7 days post fracture (dpf) showed better progression of wound healing in the Itm2a^+^ SSCs group (Figure 2, B and C). Immunofluorescence staining with an antibody against the osteoblastic marker osteopontin (OPN) in sections collected at 7 dpf showed greater colocalization of GFP and OPN in the Itm2a^+^ SSC group, indicating that Itm2a^+^ SSCs cells had stronger osteogenic potential ex vivo (Figure 2D). We also transplanted GFP^+^ cells derived from a single-cell clone underneath the renal capsule of recipient mice. The Itm2a^+^ SSC group exhibited significantly greater formation of osteoid compared with Itm2a^–^ SSCs, as assessed by μ-CT and immunofluorescence staining for OPN (Figure 2, E–G). Interestingly, Itm2a^+^ SSCs formed cortical osteoid tissue with the periosteum, showing the ability to reconstruct the cortical bone (Figure 2G). Additionally, FACS analysis showed that Itm2a^+^ SSCs were uniquely able to reconstitute their entire lineage and maintained themselves after transplantation, indicating their stemness features (Figure 2, H and I). Taken together, the Itm2a^+^ SSC sits at the apex of the hierarchal tree and is capable of self-renewal and differentiation into lineage-restricted progenitor cells (pre–bone cartilage and stromal progenitor [pre-BCSP] and BCSP).

### Itm2a-CreER labels P-SSCs.

**Figure 3:**
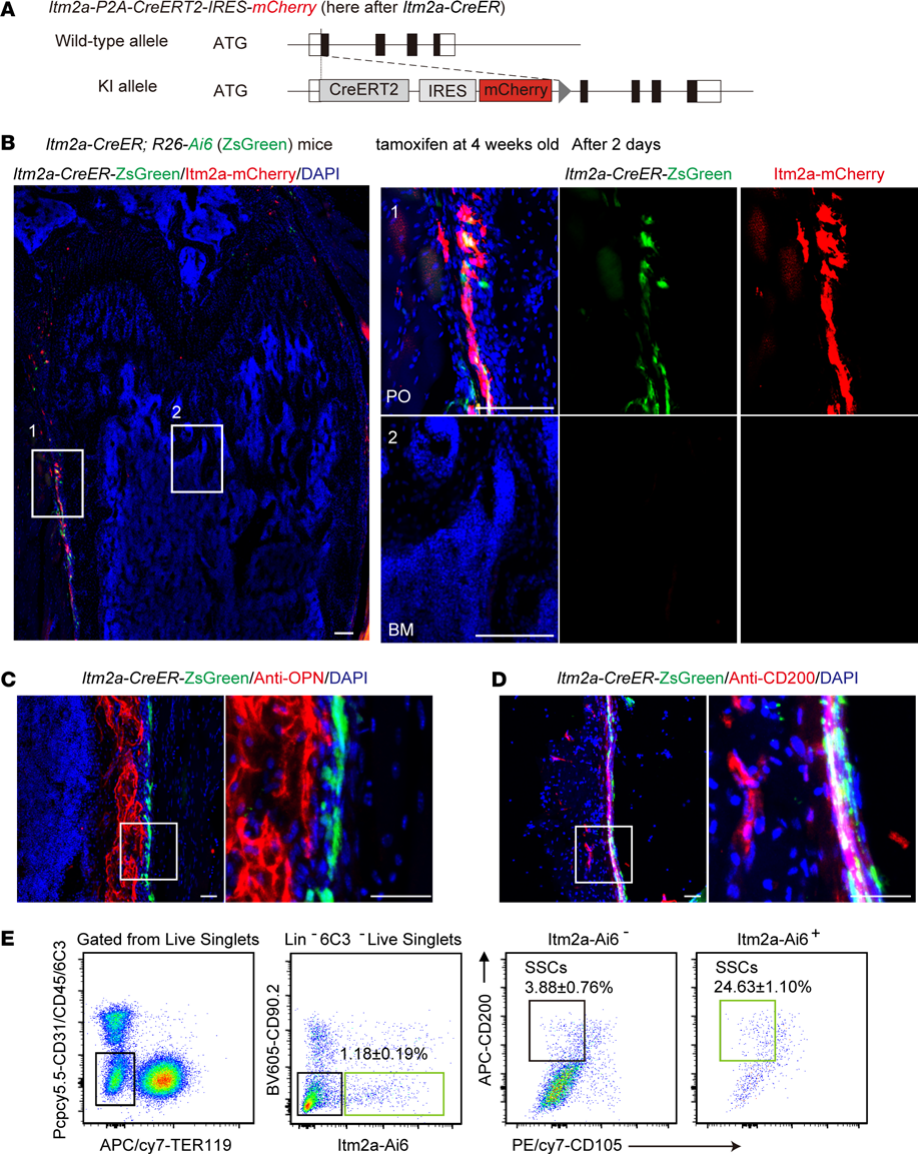
*Itm2a-CreER* labels P-SSCs. (**A**) Strategy used to construct *Itm2a-CreER-mCherry* mice. KI, knockin. (**B**) Representative confocal images of *Itm2a* lineage cells (ZsGreen^+^) and *Itm2a* expression cell (mCherry^+^) distribution in periosteal (PO) and bone marrow (BM) regions in cells from *Itm2a-CreER R26-Ai6* mice that had been tamoxifen treated at 4 weeks of age. Mice were analyzed 2 days after the treatment. Scale bars: 100 μm. (**C**) Representative confocal imaging of the osteoblast marker OPN and *Itm2a* lineage cells (ZsGreen^+^ cells) in femur sections from *Itm2a-CreER R26-Ai6* mice that had been tamoxifen treated at 4 weeks of age. Mice were analyzed 2 days after the treatment. *n* = 3 mice per condition from 3 independent experiments. Scale bars: 50 μm. (**D**) Representative confocal images of the stem cell marker CD200 and *Itm2a* lineage cells (ZsGreen^+^ ) in femur sections from mice that had been tamoxifen treated at 4 weeks of age. Mice were analyzed 2 days after the treatment. *n* = 3 mice per condition from 3 independent experiments. Scale bars: 50 μm. (**E**) Flow cytometric analysis of SSCs in ZsGreen^+^ cells from long bone digests from mice that had been treated with tamoxifen at 4 weeks of age. Mice were analyzed 2 days after the treatment. *n* = 4.

To investigate the distribution and function of Itm2a-labeled P-SSCs in vivo, we generated *Itm2a-CreERT-IRES-mCherry* mice (*Itm2a-CreER* mice) (Figure 3A). For a lineage-tracing experiment, we crossed *Itm2a-CreER* mice with *R26-LSL-Ai6* mice (hereafter referred to as *R26-Ai6*, which express ZsGreen following Cre-mediated recombination) to obtain *Itm2a-CreER R26-Ai6* mice (23). Lineage tracing showed that both *Itm2a-CreER* and Itm2a-mCherry selectively labeled periosteal cells, but few bone marrow cells, 2 days after tamoxifen injection in 4-week-old mice (Figure 3B). Flow cytometric analysis confirmed that Itm2a-ZsGreen^+^ cells and Itm2a-mCherry^+^ cells were preferentially located in the periosteum, with only trace numbers of cells present in the growth plate or bone marrow (Supplemental Figure 3, A–F). As shown in the lineage-tracing data, most of the Itm2a-mCherry^+^ cells in the periosteum were labeled by an anti-ITM2A antibody by immunostaining (Supplemental Figure 3, G and H). Itm2a-mCherry^+^ cells were preferentially located in the outer fibrous layer, which lacked periostin (Postn) expression (Supplemental Figure 4A). Itm2a-mCherry^+^ periosteal cells expressed CD200, Ctsk, Gli1, PDGFRα, Sca1, and CD51, but not the markers OPN, Osx, or CD34 (Figure 3, C and D, and Supplemental Figure 4A). After tamoxifen injection on P1, *Itm2a-CreER R26-Ai6* mice were sacrificed at P3, 1 month, and 3 months (Supplemental Figure 4B). We found that *Itm2a-CreER*–labeled cells increased gradually with postnatal bone development in the periosteal region (Supplemental Figure 4C). The percentage of Itm2a-ZsGreen^+^ cells in femoral bone also increased with bone development (Supplemental Figure 4D). The Itm2a-mCherry^+^ cells were capable of differentiation into osteoblasts, adipocytes, and chondrocytes in addition to displaying a proliferative capacity in vitro (Supplemental Figure 4E). Next, we examined the SSC lineage fraction on day 2 after tamoxifen injection into 4-week-old *Itm2a-CreER R26-Ai6* mice. The FACS data showed that the *Itm2a-CreER* also enriched a high percentage (24.6% ± 1.1%) of immunophenotypic SSCs (Figure 3E). Therefore, *Itm2a-CreER* selectively labeled periosteal cells that contained immunophenotypic P-SSCs.

### Itm2a^+^ P-SSCs generate osteoblasts, chondrocytes, and marrow stromal cells upon injury.

**Figure 4:**
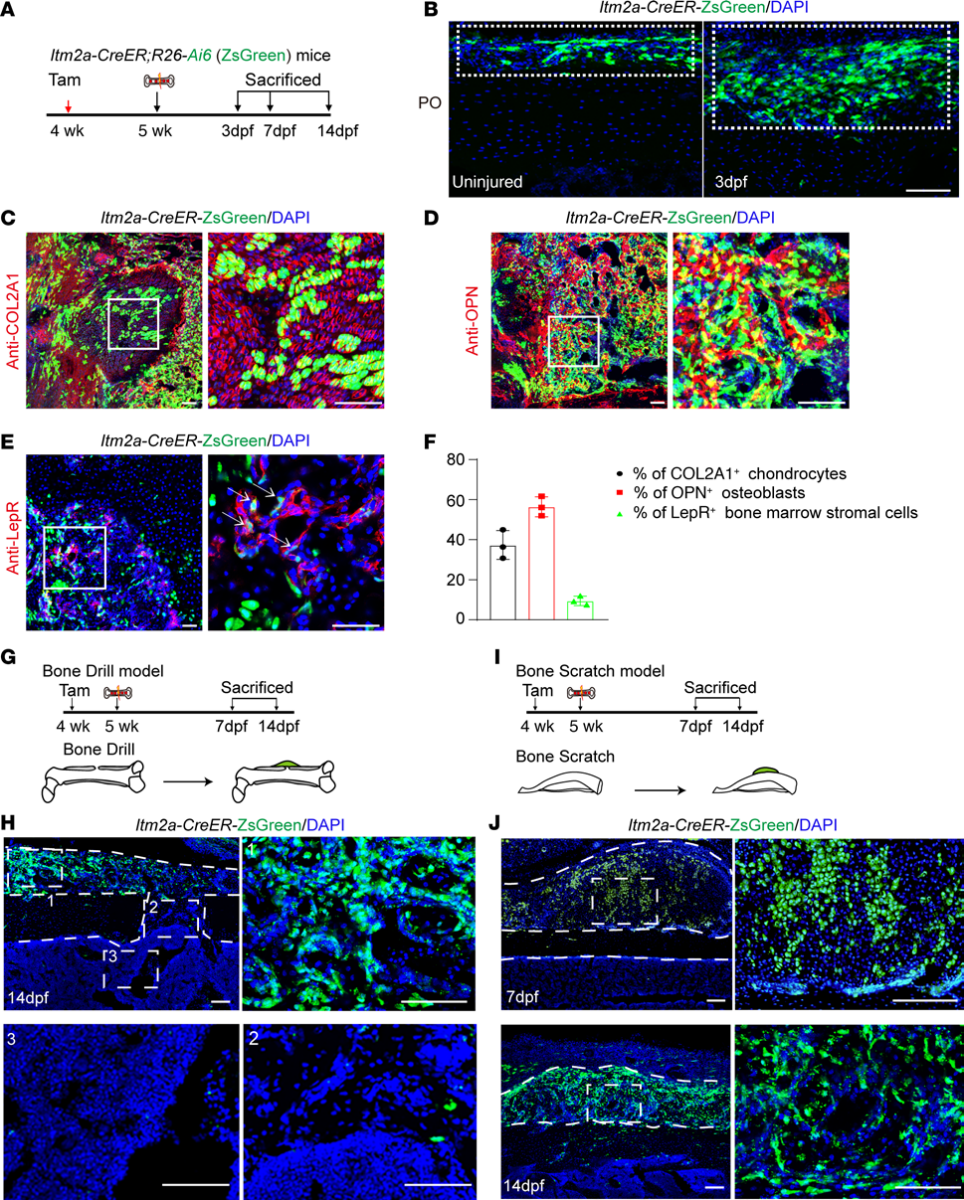
Itm2a^+^ P-SSCs generate osteoblasts, chondrocytes, and marrow stromal cells upon injury. (**A**) Experimental design for tamoxifen (Tam) induction, the bone fracture model, and tissue analysis. (**B**) Representative confocal images of *Itm2a* lineage cells (ZsGreen^+^) at the uninjured periosteum (left) and femoral fracture day-3 callus (right) in sections from *Itm2a-CreER R26-Ai6* mice. Scale bar: 100 μm. (**C**) Representative 7 dpf confocal images of *Itm2a* lineage cells (ZsGreen^+^) colocalized with chondrocytes (COL2A1) at the fractured femur site in sections from *Itm2a-CreER R26-Ai6* mice. Scale bars: 100 μm. (**D**) Representative 14 dpf confocal images of *Itm2a* lineage cells (ZsGreen^+^) colocalized with osteoblasts (OPN) at the fractured femur site in sections from *Itm2a-CreER R26-Ai6* mice. Scale bars: 100 μm. (**E**) Representative 14 dpf confocal images of *Itm2a* lineage cells (ZsGreen^+^ ) colocalized with bone marrow stromal cells (LepR) at the fractured femur site in sections from *Itm2a-CreER R26-Ai6* mice. Scale bars: 50 μm. (**F**) Percentage of *Itm2a* lineage cells (ZsGreen^+^) among COL2A1^+^ chondrocytes, OPN^+^ osteoblasts and LepR^+^ bone marrow stromal cells calculated from **C**, **D**, and **E**, respectively. *n* = 3 mice per condition. Data are presented as the mean ± SD. (**G**) Experimental design for tamoxifen induction, bone drill model, and tissue analysis. (**H**) Representative confocal images of femoral fracture sites in sections from *Itm2a-CreER R26-Ai6* mice at 14 dpf after tamoxifen treatment. *n* = 4 mice per condition from 4 independent experiments. Scale bars: 100 μm. (**I**) Experimental design for tamoxifen induction, bone scratch model, and tissue analysis. (**J**) Representative confocal images of injury sites in tibia sections from *Itm2a-CreER R26-Ai6* mice at 7 dpf and 14 dpf after tamoxifen administration. Scale bars: 100 μm. *n* = 4 mice per condition from 4 independent experiments.

To further investigate the regenerative ability of Itm2a^+^ P-SSCs in vivo, surgical fracture with internal fixation of the femur of 4-week-old *Itm2a-CreER* *R26-Ai6* mice was performed 1 week after administration of tamoxifen (Figure 4A). Three days after injury, we observed a substantial increase in ZsGreen^+^ cells in the periosteum (Figure 4B). *Itm2a* lineage cells, as defined by green fluorescence, contributed to approximately 37.4% of COL2A1^+^ chondrocytes, the majority of OPN^+^ osteoblasts (~56.6%), and approximately 9.6% of LepR^+^ stromal cells within the fracture callus (Figure 4, C–F). On the basis of these data, we concluded that *Itm2a* lineage cells regenerated the skeleton following fracture. Interestingly, *Itm2a^+^* periosteal cells mainly contributed to the formation of external callus (Supplemental Figure 5A). Next, we assessed intramembranous ossification (5) and endochondral ossification of healing bone in *Itm2a-CreER R26-Ai6* mice (Figure 4, G and I). Green fluorescent signal showed that *Itm2a* lineage cells contributed the largest proportion (>60%) of OPN^+^ osteoblasts in the external callus, but few of these lineage cells were found inside the bone after drill-hole injury (Figure 4H and Supplemental Figure 5B). Then, we generated a bone defect in the periosteum and the outer surface of cortical bone without bone marrow damage in the tibia of *Itm2a-CreER R26-Ai6* mice (Figure 4I). The majority of chondrocytes were derived from *Itm2a^+^* periosteal cells (Figure 4J and Supplemental Figure 5, C and D). Two weeks after injury, we consistently observed robust callus formation, with a greater than 60% contribution of *Itm2a^+^* cells to new osteoblasts (Itm2a^+^OPN^+^) in the injured callus (Figure 4J and Supplemental Figure 5, E and F). In addition, to compare the difference between *Itm2a^+^* and *Prrx1^+^* cells involved in fracture healing, we generated *Prrx1-CreER R26-Ai6* mice. Lineage-tracing experiments showed that *Prrx1-CreER–*labeled cells localized in the periosteum, endosteum, and bone marrow, in contrast to the specific labeling of periosteum by *Itm2a-CreER* (Supplemental Figure 6A). During fracture repair, *Itm2a* lineage cells contributed to a higher proportion of OPN^+^ osteoblasts than did *Prrx1* lineage cells at 14 dpf, as assessed by immunofluorescence staining for the osteoblastic marker OPN (Figure 4, D and F, and Supplemental Figure 6, B–D). Taken together, these data show that Itm2a labeled P-SSCs and that the activation of Itm2a^+^ P-SSCs upon cortical bone injury was sufficient to induce a periosteal response, chondrogenesis, and osteogenesis.

### Itm2a and Prrx1 lineage cells generate spatially separated subsets of chondrocytes and osteoblasts during fracture healing.

**Figure 5:**
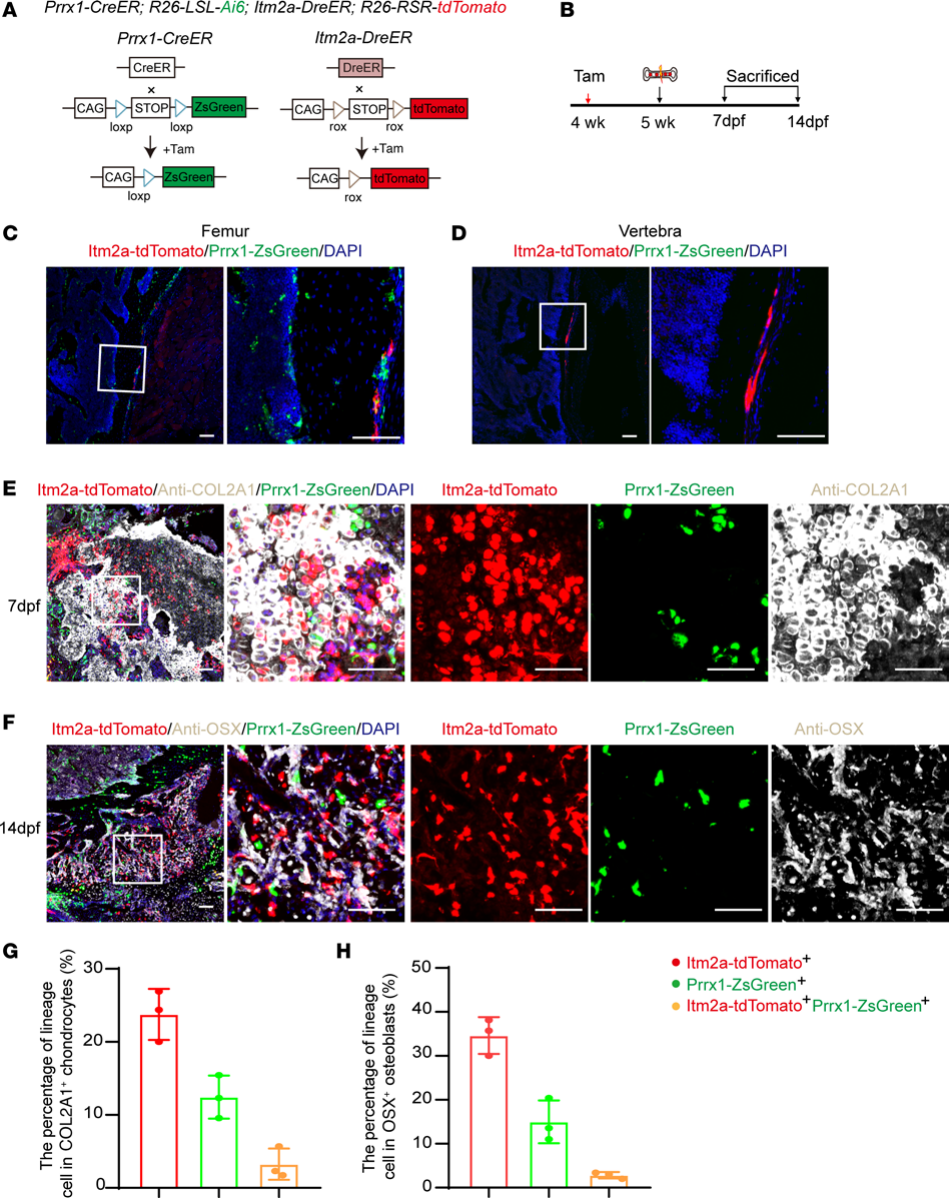
*Itm2a* and *Prrx1* lineage cells generate spatially separated subsets of chondrocytes and osteoblasts during fracture healing. (**A**) Strategy used to generate *Prrx1-CreER R26-LSL-Ai6 Itm2a-DreER R26-RSR-tdTomato* mice. (**B**) Experimental design for tamoxifen induction, bone fracture model, and tissue analysis. dpf, days post fracture. (**C** and **D**) Representative confocal images of *Itm2a* lineage cells (tdTomato^+^) and *Prrx1* lineage cells (ZsGreen^+^ cells) in femoral (**C**) and vertebral (**D**) sections from *Prrx1-CreER R26-LSL-Ai6 Itm2a-DreER R26-RSR-tdTomato* mice that had been tamoxifen treated at 4 weeks. Mice were analyzed 1 week after the treatment. *n* = 3 mice per condition from 3 independent experiments. Scale bars: 100 μm. (**E**) Representative 7 dpf confocal images of *Itm2a* lineage cells (tdTomato^+^) and *Prrx1* lineage cells (ZsGreen^+^) colocalized with chondrocytes (COL2A1) at the fractured femur site in sections from *Prrx1-CreER R26-LSL-Ai6* *Itm2a-DreER R26-RSR-tdTomato* mice. Scale bars: 100 μm. (**F**) Representative 14 dpf confocal images of *Itm2a* lineage cells (tdTomato^+^) and *Prrx1* lineage cells (ZsGreen^+^) colocalized with osteoblasts (OSX^+^) at the fractured femur site in sections from *Prrx1-CreER R26-LSL-Ai6 Itm2a-DreER R26-RSR-tdTomato* mice. Scale bars: 100 μm. (**G** and **H**) Percentage of 3-cell populations among COL2A1^+^ chondrocytes and OSX^+^ osteoblasts calculated from **E** and **F**, respectively. *n* = 3 mice per condition. Data are presented as the mean ± SD.

Given that both *Prrx1* lineage cells and *Itm2a* lineage cells contributed to the bone fracture healing, we generated *Prrx1-CreER R26-LSL-Ai6 Itm2a-DreER R26-RSR-tdTomato* mice to define the relationship between them in vivo. In this line, the *Itm2a* lineage cells express tdTomato and the *Prrx1* lineage cells express ZsGreen (Figure 5A). Surgical fracture with internal fixation of the femur of *Prrx1-CreER* *R26-LSL-Ai6* *Itm2a-DreER R26-RSR-tdTomato* mice was performed 1 week after administration of tamoxifen to 4-week-old mice (Figure 5B). Interestingly, Itm2a^+^Prrx1^+^ (yellow) cells only accounted for a small proportion in the femur periosteum, which indicated that Itm2a^+^ cells may have had only a modest overlap with Prrx1^+^ cells (Figure 5C). Itm2a also labeled periosteal cells in vertebra, whereas Prrx1 labeled few cells in this region (Figure 5D). Furthermore, *Itm2a* lineage cells contributed more chondrocytes (Figure 5, E and G) and osteoblasts (Figure 5, F and H) in the callus than did *Prrx1* lineage cells during the bone fracture healing process. Surgical fracture with internal fixation and bone drill-hole of the femur of *Prrx1-CreER R26-LSL-Ai6* *Itm2a-DreER R26-RSR-tdTomato* mice was also performed 8 weeks after administration of tamoxifen to P1 mice. The results were consistent with those seen after induction at 4 weeks (Supplemental Figure 7, A–H). The above data demonstrate that there were differences between the 2 cell populations.

### BMP signaling is critical for Itm2a^+^ P-SSCs and contributes to bone fracture healing.

**Figure 6:**
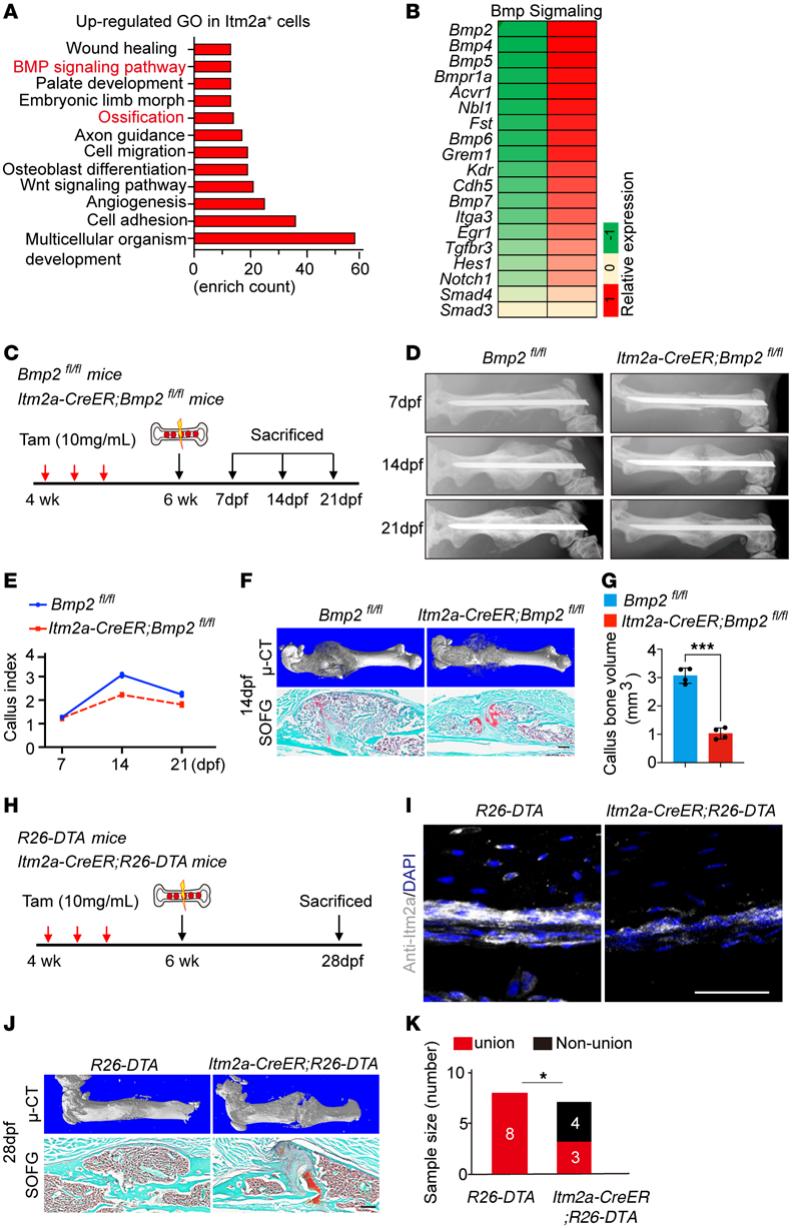
BMP signaling is critical for the Itm2a^+^ P-SSC contribution to bone fracture healing. (**A** and **B**) Bulk RNA-Seq data from Lin^–^Itm2a^–^ and Lin^–^Itm2a^+^ periosteal cells isolated from 4-week-old WT mice. GO plot (**A**) and heatmap (**B**) show that BMP signaling was enriched in Lin^–^Itm2a^+^ periosteal cells. (**C**) Experimental design for tamoxifen induction, the bone fracture model, and tissue analysis of *Itm2a-CreER Bmp2^fl/fl^* and control mice. (**D**) Analysis by x-ray of fractured femurs in *Itm2a-CreER Bmp2^fl/fl^* mice and control mice at 7, 14, and 21 dpf. *n* = 4 mice per genotype per time point. (**E**) Callus index calculated from x-ray data showed callus formation at 7, 14, and 21 dpf in mutant and control mice. *n* = 4 mice per group. Data are presented as the mean ± SD (*n* = 4). (**F**) μ-CT (upper) and SO/fast green (SOFG) (lower) staining analysis of fractured femurs in *Itm2a-CreER Bmp2^fl/fl^* mice and control mice at 14 dpf. *n* = 4 mice per genotype. Scale bar: 100 μm. (**G**) Bone volume quantification showed callus formation at 14 dpf in mutant and control mice. *n* = 4 mice per group. Data are presented as the mean ± SD (*n* = 4). ***P < 0.001, by unpaired, 2-tailed *t* test. (**H**) Experimental design for tamoxifen induction, the bone fracture model, and tissue analysis of *Itm2a-CreER* *R26-DTA* and *R26-DTA* mice. (**I**) Confocal images of *Itm2a^+^* cell (anti-*Itm2a* staining) at the periosteal region from *R26-DTA* (left) and *Itm2a-CreER R26-DTA* (right) mice. Scale bar: 50 μm. (**J**) μ-CT (upper) and SOFG (lower) staining analysis of fractured femurs in *R26-DTA* and *Itm2a-CreER* *R26-DTA* at 14 dpf. Scale bar: 100 μm. (**K**) Non-union percentage calculated from *R26-DTA* and *Itm2a-CreER R26-DTA* mice at 28 dpf. *P = 0.025, by Fisher’s exact test.

BMP2 is one of the crucial components of the BMP signaling cascade in human fracture callus, and we found that the wound-healing and BMP signaling pathways were enriched in Lin^-^Itm2a^+^ cells (Figure 6, A and B). Ossification and angiogenesis signaling pathways, which are essential for bone fracture healing, were also enriched in Lin^-^Itm2a^+^ cells (Figure 6A). Bmp2 is indispensable for skeletal development and bone fracture healing (24). However, whether BMP2 is necessary in P-SSCs has not, to our knowledge, been tested yet. Therefore, we crossed *Itm2a-CreER Bmp2^fl/+^* mice with *Bmp2^fl/fl^* mice to obtain *Itm2a-CreER Bmp2^fl/fl^* mice (Figure 6C). After 3 consecutive tamoxifen injections into 4-week-old female mice, *Itm2a-CreER Bmp2^fl/fl^* mice showed a delayed endochondral ossification in bone fracture healing at 14 dpf (Figure 6, D–G).

To further investigate the function of Itm2a^+^ P-SSCs in vivo, we crossed *Itm2a-CreER* mice with *R26-DTA* mice to ablate *Itm2a-CreER*–expressing cells after 3 consecutive tamoxifen injections into 4-week-old female mice (25). We performed identical unilateral femoral fracture surgery (Figure 6H). As expected, *Itm2a-CreER* *R26-DTA* mice showed decreased Itm2a^+^ cells and increased rates of fracture nonunion at 28 dpf as assessed by μ-CT, safranin O (SO), and fast green staining (Figure 6, I–K). These data indicate that Itm2a^+^ P-SSCs contributed functionally to bone fracture healing.

### ITM2A enriches P-SSCs in human periosteum samples.

**Figure 7:**
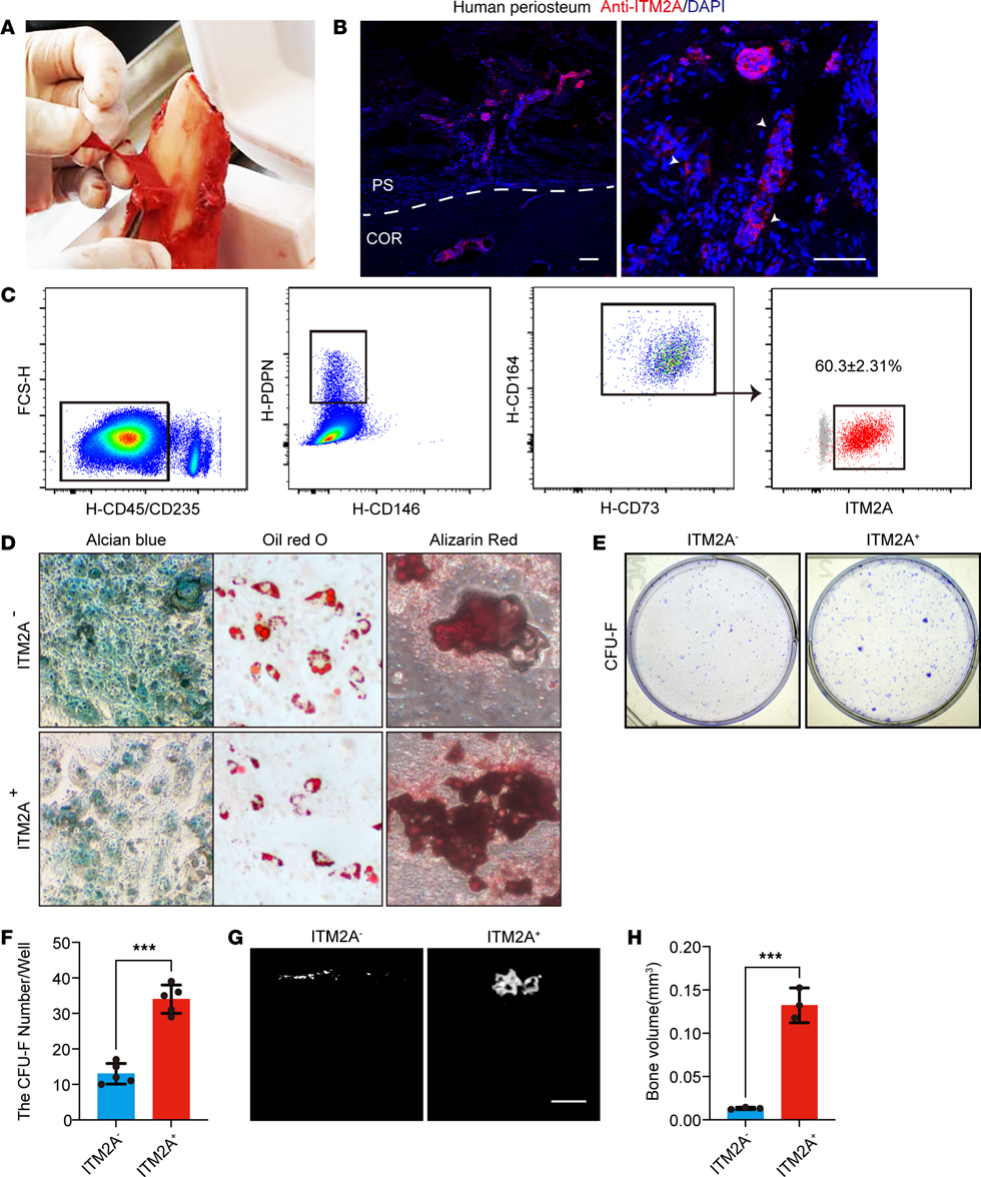
ITM2A enriches P-SSCs in human periosteum samples. (**A**) Representative image of a human periosteum specimen. (**B**) Immunostaining for ITM2A in human fibula sections. Arrows indicate ITM2A^+^ cells in the periosteal region. Scale bars: 100 μm. COR, cortical bone; PS, periosteum. (**C**) Flow cytometric analysis of the expression of human (H) SSC markers in ITM2A^+^ cells digested from human fibular periosteum. FSC-H, forward scatter height. (**D**) In vitro 3-lineage differentiation of sorted Lin^–^ITM2A^–^ and Lin^–^ITM2A^+^ human periosteal cells. Representative images of Alcian blue staining, Oil Red O staining, and alizarin red staining are shown. Original magnification, ×20. (**E** and **F**) A CFU-F assay of Lin^–^ITM2A^–^ and Lin^–^ITM2A^+^ cells from human fibular periosteum was performed. (**E**) Representative CFU-F image and (**F**) the number of CFU-U per well. *n* = 5 independent experiments per condition. Data are presented as the mean ± SD. ***P < 0.001, by unpaired *t* test. (**G** and **H**) Kidney capsule transplantation assay of Lin^–^ITM2A^–^ and Lin^–^ITM2A^+^ cells from human fibular periosteum. (Representative μ-CT images (**G**) and bone volume quantification (**H**). *n* = 4 independent experiment per condition. Data are presented as the mean ± SD. ***P < 0.001, by unpaired *t* test.

To explore the translational implications of our findings, human periosteum tissue from the femur was sectioned and stained, showing the expression of ITM2A (Figure 7, A and B). Additionally, FACS analysis of primary cells isolated from collagenase-digested human periosteum tissue showed that Lin^–^ITM2A^+^ cells had higher numbers of cells expressing human SSC–defining markers (PDPN^+^CD146^–^CD73^+^CD164^+^) than did Lin^–^ITM2A^–^ cells (Figure 7C). FACS-sorted Lin^–^ITM2A^+^ cells showed a stronger CFU-F ability (Figure 7, D and E). These human ITM2A^+^ periosteal cells were multipotent, as they underwent osteogenic, adipogenic, and chondrogenic differentiation in vitro (Figure 7F). We then performed a bone organoid assay using human Lin^–^ITM2A^+^ and Lin^–^ITM2A^–^ cells. The sorted cells were transplanted into the kidney capsule of immunocompromised mice. We observed that the Lin^–^ITM2A^+^ cells formed larger bone organoids than did Lin^–^ITM2A^–^ cells (Figure 7, G and H). These results suggest that human ITM2A^+^ cells may be involved in the repair of human bone.

### Itm2a^+^ P-SSCs decrease in aged mice.

Loss of skeletal stem cell function may be related to the impaired bone healing in the setting of aging (26). We conducted lineage-tracing and femoral bone fracture experiments in adult (12 weeks of age) and aged (18 months of age) *Itm2a-CreER R26-LSL-Ai6* mice (Supplemental Figure 8A). The aged mice showed delayed fracture healing, as reported by previous studies (26, 27) (Supplemental Figure 8B). Itm2a^+^ cells decreased significantly in the periosteum of aged mice (Supplemental Figure 8, C and D). The Itm2a^+^ cell–derived OPN^+^ osteoblasts decreased at 14 dpf in aged mice (Supplemental Figure 8, E and F). These data support the idea that Itm2a^+^ cells continue to participate in fracture repair in adult and aging mice.

## Discussion

Here, we used single-cell transcriptomics to identify an Itm2a^+^ cell population as a potential marker of P-SSCs. Lineage-tracing experiments showed that these cells can proliferate and give rise to osteoblasts, chondrocytes, and marrow stromal cells upon injury. We also demonstrated the relationship between Prrx1^+^ and Itm2a^+^ cells responding to bone fracture healing by using a dual-recombinase system (28, 29). Interestingly, our lineage-tracing experiments showed that Itm2a^+^ cells were mainly distributed in the outer fibrous layer of the periosteum and that these cells gave rise to an approximately 37% presence of chondrocytes in the fracture callus during fracture healing, suggesting that there was a higher proportion of endochondral ossification in the fracture-healing process of other stem cell populations in other parts of the periosteum. Itm2a^+^ P-SSCs were also present in the human periosteum. Thus, our study identified a membrane protein marker labeling P-SSCs, providing an attractive target for drug and cellular therapy for skeletal disorders.

The periosteum is critical for bone repair, and previous studies have shown that different marker genes could label periosteal cells contributing to bone regeneration (6–13, 15). Among these markers, many of them label cells in the bone marrow and are difficult to distinguish from periosteal cells. Given the importance of both bone marrow cells and periosteal cells for fracture repair, it was difficult to confirm whether some of the signaling pathways and transcription factors that have been found to be important for fracture repair were working through periosteal stem/progenitor cells (12, 30). *Itm2a-CreER* can selectively label P-SSCs and may be a good model for the study of the periosteum.

In the bulk sequencing data, we found that Itm2a^+^ P-SSCs were enriched for the expression of bone morphogenetic protein (BMP) pathway–related genes. *Bmp2* has been reported to be critical for fracture repair (31). Consistent with our expectations, we observed impaired fracture repair in mice with *Bmp2* deletion in the *Itm2a*-expressing lineage. Mice lacking the ability to produce BMP2 in the *Prrx1* lineage exhibit spontaneous fractures that do not resolve over time (24). Interestingly, *Osx-Cre* *Bmp2^fl/fl^* mice have no fracture repair phenotype (32). These data indicate that *Itm2a^+^* cells are probably a source of BMP2 during fracture healing. In addition, *Itm2a^+^* cells were enriched in Wnt signaling. Wnt signaling may be critical for Itm2a^+^ P-SSCs to participate in fracture repair, and further experimental verification is needed (33).

Researchers have been working to identify human SSCs (2, 4, 10). Chan et al. identified human SSCs from the growth plate using combined cell-surface markers PDPN^+^CD146^–^CD73^+^CD164^+^ (4). Cells sorted by PDPN^+^CD146^–^CD73^+^CD164^+^ can label human periosteal cells that respond to injury. Interestingly, ITM2A enriched human SSCs and Lin^–^ITM2A^+^ periosteal cells displayed stronger stemness features than did Lin^–^ITM2A^–^ cells, which prompted us to conclude that the subpopulation of cells represented bona fide human P-SSCs.

In summary, we identified ITM2A as a marker of P-SSCs participating in bone development and regeneration. As Itm2a itself is a membrane protein and anti-ITM2A antibody could be used to isolate human ITM2A^+^ P-SSCs, it is possible that ITM2A^+^ P-SSCs could be used to advance healing in patients with incomplete fracture repair, as the loss of skeletal stem cell function may be related to the impaired bone healing (26). Identification of Itm2a^+^ P-SSCs will both aid in determining the mechanism of existing skeletal therapies acting on the periosteum and also potentially enable cellular therapies for skeletal disorders by identifying a periosteal cell with a high degree of reparative function.

## Methods

### Sex as a biological variable.

Our study examined male and female animals, and similar findings are reported for both sexes.

### Animals.

*Itm2a-CreERT2-IRES-*mCherry and *Itm2a-DreER* mice were generated with the assistance of Biocytogen. *Prrx1-Cre* was purchased from The Jackson Laboratory. *Prrx1-CreER* mice were provided by Baojie Li (Shanghai Jiao Tong University, Shanghai, China) (34). *Rosa26-LSL-tdTomato (R26-Ai9)* mice were provided by Zilong Qiu (Shanghai Jiao Tong University, Shanghai, China) (23). *Bmp2^fl/fl^* mice, *Rosa26-LSL-Ai6 (R26-Ai6)* reporter mice, and *Rosa26-DTA (R26-DTA)* mice were provided by Aria Zeng (Center for Excellence in Molecular Cell Science, Shanghai, China) (23). CreER-expressing mice were induced by intraperitoneal injection every other day of 2 mg tamoxifen dissolved in corn oil and repeated 3 times.

All mice were maintained on a C57/BL6 background, and sex-matched littermate controls were used in all analyses. All animals were housed under specific pathogen–free conditions.

### Single-cell sequencing of sorted periosteal cells from Prrx1-Cre R26-Ai9 mice.

Sorted tdTomato^+^ cells from *Prrx1-Cre R26-Ai9*^+^ periosteal cells obtained from the mouse hind limb at the indicated developmental stage (4 weeks) were subjected to 10X Chromium Single Cell 3′ Solution (version 2) library preparation according to the manufacturer’s instructions (10X Genomics). Library sequencing was performed with an Illumina HiSeq 2500 sequencer to a depth of 100 million reads in each sample. Reads were converted to the fastq format using mkfastq in Cell Ranger 2.1.0 (10X Genomics). Reads were then aligned to the mouse reference genome (mm10, Ensembl annotation release 91), including the additional sequence and feature annotation for tdTomato. Alignment was performed using the count command of Cell Ranger 2.1.0 (10X Genomics). Primary analysis, quality control filtering (gene count per cell, unique molecular identifier counts per cell, and percentage of mitochondrial transcripts), clustering, cell-cycle phase scoring based on canonical markers and regression analysis, identification of cluster markers, and visualization of gene expression were performed using the Seurat (version 2.3) package for R.

The construction of single-cell trajectories, identification of differentially expressed genes as a function of pseudotime, and clustering of genes by pseudotemporal expression patterns were performed using the Monocle 2 package for R. Pseudotime calculations were performed on the top 1,000 differentially expressed genes between clusters. The Database for Annotation, Visualization and Integrated Discovery (DAVID) Functional Annotation Bioinformatics Microarray Analysis Test (https://david.ncifcrf.gov) was used for the Gene Ontology (GO) enrichment analysis of biological processes.

### RNA-Seq.

For bulk RNA-Seq of Lin^–^Itm2a^–^ and Lin^–^Itm2a^+^ periosteal cells, a single periosteal cell suspension was isolated from 4-week-old male C57 mice. To sort Lin^–^Itm2a^–^ and Lin^–^Itm2a^+^ periosteal cells, the cell suspension was incubated with anti-Itm2a and anti-lineage (CD31, CD45, and TER119) antibodies for 30 minutes, and after washing with PBS, cell sorting was conducted using Aria Sorp (BD Bioscience). Cells were directly sorted into TRIzol LS buffer (Invitrogen, Thermo Fisher Scientific, 10296-028), held in 1.5 mL RNase-free EP tube, and then approximately 100,000 cells were collected for each gate. RNA-Seq library preparation was conducted according the VAHTS Universal V6 RNA-Seq Library Prep kit manual (Vazyme, NR605-01). The size of the libraries was selected using DNA Clean beads (Vazyme, N411-01), with an average size of 300 bp. The libraries were sequenced using the Illumina HiSeq 4000 system (paired-end 50 bp). The RNA-Seq reads were aligned to the GRCm38.98 *Mus musculus* reference genome with STAR (version 2.7.2a) using a supplied set of known transcripts in GTF format (RefSeq GRCm38.98; *Mus musculus*, Ensembl). Differentially expressed genes were calculated by fold change with a cutoff (log fold change >1.5, and reads per kilobase per million mapped reads [RPKM] > the lower quartile), and RPKM values were calculated using a custom R script.

### Histological analysis.

For frozen sectioning, freshly dissected tibiae were fixed with 4% paraformaldehyde (PFA) for 48 hours at 4°C, decalcified in 15% EDTA, and dehydrated in 30% sucrose for 48 hours. The tissue samples were then embedded in OCT (18) compound (Tissue-Tek, 4583) and sectioned at 12–16 μm thickness using a Leica CM3050S cryostat. Sections were dewaxed and rehydrated and then stained with SO.

### Immunohistochemistry and immunofluorescence staining.

For immunofluorescence staining, frozen sections were air dried, rehydrated with PBS, and then blocked and permeabilized with 3% BSA and 0.2% Triton X-100 in PBS at room temperature for 45 minutes. Sections were probed with the following primary antibodies overnight at 4°C: rabbit anti-Itm2a (Invitrogen, Thermo Fisher Scientific, PA5-49585, 1:200); goat anti-OPN (R&D Systems, AF808, 1:500); mouse anti-Col2a1 (Abcam, Ab185430, 1:200); goat anti-LepR (R&D Systems, BAF497, 1:200); rabbit anti-Ki67 (Abcam, ab15580, 1:200); rabbit anti-Runx2 (Abcam, ab114133, 1:200); mouse anti-Postn (R&D Systems, AF2955, 1:200); rat anti-PDGFRα (BioLegend, 135910,1:200); rabbit anti-Gli1 (Abcam, ab273018, 1:200); mouse anti-Ctsk (Santa Cruz Biotechnology, sc-48353, 1:200); rat anti-Sca1 (Invitrogen, Thermo Fisher Scientific, 17-5981-81, 1:200); rat anti-CD34 (BioLegend, 128618, 1:200); and rat anti-CD51 (BioLegend, 104103, 1:200). Fluorescence dye–labeled secondary antibodies, including donkey anti–rabbit Alexa Fluor 647 (Molecular Probes, A21206, 1:1,000), donkey anti–goat Alexa Fluor 647 (Molecular Probes, A11055, 1:1,000), donkey anti–mouse Alexa Fluor 647 (Molecular Probes, A21202, 1:1,000), and goat anti–rat Alexa Fluor 647 (Molecular Probes, A21247, 1:1,000), were incubated with sections for 1 hour at room temperature after washing. Nuclei were counterstained with DAPI (MilliporeSigma, D9542). Sections were mounted using fluorescence mounting medium (Dako, S3023).

### Flow cytometry.

Cell sorting was performed with an Arial Sorp Cell Sorter (BD Biosciences). Adhesive cells and debris were excluded via forward scatter (FSC) and side scatter (SSC) profiles. For the analysis of Ai6^+^ cells among periosteal cells from *Itm2a-CreER R26-Ai6* mice, equal numbers of cells were added into each individual tube containing different antibodies for immunostaining using a previously described method (35). RBCs were first removed by incubating the samples with RBC lysis buffer (Beyotime, C3702). Then, the cells were stained with eFluor 450–conjugated anti-CD31 (eBioscience, 48-0311-80), PerCP/Cy5.5-conjugated anti-CD45 (BioLegend, 103132); APC/Cy7-conjugated anti–mouse TER-119 (BioLegend, 116223); PerCP/Cy5.5-conjugated anti-mouse 6C3/Ly-51 (BioLegend, 108315); Brilliant Violet 605–conjugated anti–mouse CD90.2 (BioLegend, 140317); PE/Cy7-conjugated anti–mouse CD105 (BioLegend, 120409); APC-conjugated anti–mouse CD200 (BioLegend, 123809); Pacific blue–conjugated anti–human CD235 (BioLegend, 306612); Pacific blue–conjugated anti–human CD45 (BioLegend, 304029); PE/Cy7-conjugated anti–human CD146 (BioLegend, 342010); BV510-conjugated anti–human podoplanin (BD Biosciences, 747635); PE-conjugated anti–human CD164 (BioLegend, 324808); FITC-conjugated anti–human CD73 (BioLegend, 344016); and Cy3-conjugated anti-rabbit (Jackson ImmunoResearch, 711-165-152, 1:1,000) antibodies. After washes with PBS, flow cytometric analysis was conducted using a Beckman CytoFLEX FCM, and the data were analyzed with FlowJo software.

### Analysis of human periosteal cell properties.

All specimens were collected from the fibula resected for a non-neoplastic medical indication, and the patients (patient 1, 25 years of age, female; patient 2, 45 years of age, female) did not have comorbidities that would affect fracture healing. Cells were digested using the same method as mouse samples. For the analysis of ITM2A^+^ cells from human periosteum, equal numbers of cells were added to each individual tube containing different antibodies for immunostaining using a previously described method (35). After washes with PBS, flow cytometric analysis was conducted using a Beckman CytoFLEX FCM, and the data were analyzed with FlowJo software.

### Isolation and culturing of periosteal cells.

Femurs and tibiae were thoroughly cleaned to remove all soft tissue. Then, the epiphysis and metaphysis region were coated with low-melting-point agarose (10% in TAE buffer). The agarose-coated femurs and tibiae were preserved in ice-cold PBS and then digested with 1 mg/mL collagenase (MilliporeSigma, C0130), 2 mg/mL dispase II (MilliporeSigma, D4693), and 2% penicillin/streptomycin in minimum essential medium α (α-MEM) (Corning, 10-022-CVR) at 37°C for 30 minutes. The cell suspension was passed through a 70 μm nylon mesh (BD Falcon, BD Biosciences, 352350) and plated in 1 well of a 6-well plate in growth medium (α-MEM containing 10% FBS) (Ausbian, VS500T) and 1% penicillin/streptomycin (Gibco, Thermo Fisher Scientific, 10378-016). Cells were replated in a 6 cm dish when they reached 80%–90% confluence.

### Colony formation assay and in vitro analysis of multipotent differentiation.

Itm2a^+^ periosteal cells labeled with other markers were sorted by culturing primary isolated periosteal cells for 7 days after sorting with an anti-Itm2a antibody and then digested them into a single-cell suspension. Cell sorting was performed with an Arial Sorp Cell Sorter (BD Biosciences). Adhesive cells and debris were excluded via FSC and SSC profiles.

For the colony formation assay, single sorted Itm2a^+^ periosteal cells were cultured for 14 days. In vitro analyses of multipotent osteogenic, adipogenic, and chondrogenic differentiation were performed as previously described (35).

The sorted cells were plated at a density of 1,000 cells per 10 cm dish and allowed to form individual colonies. The extracted cells from clones were regrown for 7 days and then induced to differentiate into osteoblasts, adipocytes, and chondrocytes.

For osteogenic differentiation, cells that were sorted by flow cytometry were plated in a 96-well plate. Cells were cultured in osteoblast induction medium (α-MEM containing 10% FBS), 5 mM β-glycerophosphate (MilliporeSigma, G9422), 50 μg/ml l-ascorbic acid (MilliporeSigma, A5960), and 1% penicillin/streptomycin (Gibco, Thermo Fisher Scientific, 15140-122)). The medium was changed every 3 days. After 21–28 days of induction, cells were fixed with 10% neutral buffered formalin (MilliporeSigma, HT501320) and stained with the alizarin red solution according to the manufacturer’s instructions.

Adipogenic differentiation was similarly conducted with sorted cells as described above. In brief, cells were allowed to differentiate in adipogenic differentiation medium. Fresh medium was added every 2–3 days for a total of 14–20 days. Medium was removed, and cells were washed with PBS and fixed with 4% PFA for 30 minutes at room temperature. Cells were rinsed again with PBS and stained for 30 minutes with Oil Red O working solution (3:2 dilution with water). Cells were then observed under a light microscope after 4–5 washes with PBS.

For chondrogenic differentiation, a micromass culture method was used to determine the chondrogenic differentiation ability. The sorted Itm2a^+^ periosteal cells were plated as a 10 μL micromass drop in a culture well of a 24-well plate and incubated with 5% CO_2_ at 37°C for 2 hours to allow cell attachment. Then, 1 mL chondrocyte induction medium (α-MEM containing 10% FBS), 1% ITS (Cyagen, ITSS-10201-10), 10 ng/mL TGF-β3 (Peprotech, 100-36E), 100 nM dexamethasone (MilliporeSigma, D1756), 1 mM sodium pyruvate (MilliporeSigma, 25-000-CIR), 40 μg/mLl proline (MilliporeSigma, P5607), 50 μg/mL l-ascorbic acid 2-phosphate (MilliporeSigma, A8960), and 1% penicillin/streptomycin) was added and incubated for 4 days. The micromass was acidified with 0.1 N HCl and stained with 1% Alcian blue (MilliporeSigma, A5268).

### Kidney capsule transplantation model.

Briefly, 8- to 10-week-old nude mice were anesthetized, and the left flank and abdomen were shaved before sterilization of the surgical site. The kidney was externalized through a 1 cm incision, and a 2 mm pocket was created in the renal capsule. A 5 μL Matrigel plug (Corning, 356231) containing approximately 10,000 cells digested from single-cell–derived clones were implanted underneath the capsule, and the hole was sealed using medical chitosan liquid dressing before re-placing the kidney in the body cavity. The recipient mice used in these experiments were syngeneic with the donor mice. All transplantation studies using Itm2a^+^ cells were conducted in WT hosts. Animals were euthanized with CO_2_ after 8 weeks. After death, the kidneys were fixed with 4% PFA for 5 hours, and bone formation was detected by sectioning tissues.

### Mouse femoral bone fracture model.

Five- to 6-week-old male and female mice were used to establish the model. The mice were anesthetized using chloral hydrate. The patella was dislocated laterally to expose the femoral condyles, an intramedullary pin was inserted to stabilize the femur, and the patella was then relocated. The fracture was generated using a dentist’s microdrill at the midpoint of the femur. The muscles were repositioned, and the skin was closed using a 6/0 nylon suture. Fracture repair was followed radiographically using an MX2 x-ray system (6 seconds at 932 kV; MX2 Radiation).

### Bone-drill and bone scratch model.

The model of bone repair by intramembranous ossification was established with a drill-hole injury in the middle of the femur. A skin incision was made at the middle of the femur. Blunt dissection of the subcutaneous tissue was performed until the periosteum was exposed. A needle of 0.7 mm in diameter was used to drill a hole into the anterior cortices. Afterward, the subcutaneous tissue was repositioned, and the skin was closed using a 6/0 nylon suture.

The model of bone repair by endochondral ossification was established with a scratch of the periosteum in the middle of the anteromedial tibia. After the mice were anesthetized and the periosteum was exposed, a needle tip was used to scratch the periosteum lengthwise in the middle of the left anteromedial tibia. The length of the scratch was 0.5–1 cm. Afterwards, the subcutaneous tissue was repositioned, and the skin was closed using a 6/0 nylon suture.

### Radiographic assessments.

For the x-ray image analysis, mice were euthanized with CO_2_, followed by the removal of skin and internal organs. The skeletons were then fixed with 70% ethanol and analyzed by whole-body x-ray using an Eagle III Microspot X-ray Fluorescence instrument (Exda Inc.) and a Faxitron SR radiograph digital imaging system.

For the μ-CT analysis, femurs isolated from age- and sex-matched mice were fixed with 70% ethanol and scanned using a SkyScan1272 at 20 μm resolution for qualitative analysis or at 10 μm resolution for quantitative analysis. 3D images were reconstructed using a fixed threshold.

### Statistics.

Data were generated from independently obtained datasets and are presented as the mean ± SD. Two groups were compared using either paired or unpaired, 2-tailed *t* tests. Fisher’s exact test was used to determine whether there was a significant association between Itm2a^+^ cell depletion and nonunion in Figure 6K. *P* values of less than 0.05 were considered to indicate statistically significant differences.

### Study approval.

Animal experiments were approved and conducted in full compliance with protocols approved by the Institutional Animal Care and Research Advisory Committee of the Shanghai Institute of Biochemistry and Cell Biology (SIBCB). This study complied with all relevant ethics regulations for animal testing and research. All animal experiments were performed in the Animal Facility of the SIBCB and according to the protocol (approval no. SIBCB-NAF-14-001-S350-019) authorized by the IACUC of the SIBCB and the Chinese Academy of Sciences. For the harvesting of human periosteum samples, the specimens were harvested from 3 different patients with autogenic-free fibular grafting at Shanghai Jiao Tong University Affiliated Sixth People’s Hospital according to the protocol (no. 2020-118) authorized by the Ethics Committee of Shanghai Jiao Tong University Affiliated Sixth People’s Hospital.

### Data availability.

The scRNA-Seq and group RNA-Seq datasets generated in this study are publicly available in the Genome Sequence Archive (GSA) (https://ngdc.cncb.ac.cn/gsa) under accessions numbers CRR547971 and CRA007831. All data values reported in this work are available in the Supporting Data Values file.

## Supplementary Material

Supplemental data

Supporting data values

## Version 1 09/03/2024

Electronic publication

## Version 2 12/10/2024

added missing supplemental data file
