# Supplementary material for: Itm2a expression marks periosteal skeletal stem cells that contribute to bone fracture healing
Source: J Clin Invest. 2024 Sep 3;134(17):e176528. doi: 10.1172/JCI176528 (PMC11364384; doi:10.1172/JCI176528)
Supplement: Supplemental data [file jci-134-176528-s008.pdf]

Fig. S1 Itm2a expression pattern in *Prrx1* lineage periosteal cells scRNA-seq data, related to Figure 1.

(A) Cluster distribution of periosteal stem/progenitor cells.

(B) Feature plots of *Prrx1*, *Ctsk, Gli1, LepR* and *Clec11a* expression in *Prrx1-Cre* Ai9 cells.

(C) Bubble plot showing the top 14 marked genes in cluster 1.

Fig. S2 The membrane protein Itm2a-expressing periosteal cells generate CFU-F and are multipotent, related to Figure 2.

(A) Confocal images of plasmid transfected 293 cells (GFP^+ cells) colocalized with a cell membrane marker (Dil) to show the membrane distribution of ITM2A protein.

(B&C) Representative images (C) and statistic data (D) of CFU-F assay of Lin^-Itm2a^- and Lin^-Itm2a^+ cells from 4-week-old WT mice (D) (n = 4, from 4 independent experiments). Unpaired t test, ***P < 0.001.

(D) *In vitro* three-lineage differentiation of sorted Lin^-Itm2a^- and Lin^-Itm2a^+ mice periosteal single cell-derived clones. Representative images of Alcian blue staining (left panel), oil red staining (middle panel) and alizarin red staining (right panel) are shown.

Fig. S3 The verification of *Itm2a-CreER; R26-Ai6* mice, related to Figure 3.

(A) Flow cytometry analysis revealed that Itm2a-$mCherry^+$ cells in the periosteum (left), the growth plate (middle) and bone marrow (right). Mice were analyzed 2 days after the tamoxifen treatment at 4 weeks of age.

(B) The percentage of Itm2a-$mCherry^+$ cells among Lin^- cells calculated from Figure S3A. n = 3 mice per condition from 3 independent experiments.

(C) Flow cytometry analysis revealed that Itm2a-$ZsGreen^+$ among Itm2a-$mCherry^+$ cells in the periosteum (left), the growth plate (middle) and bone marrow (right). Mice were analyzed 2 days after the tamoxifen treatment at 4 weeks of age.

(D) The percentage of Itm2a-$ZsGreen^+$ cells among Itm2a-$mCherry^+$ cells calculated from Figure S3C. n = 3 mice per condition from 3 independent experiments.

(E) Flow cytometry analysis revealed that Itm2a-mCherry$^+$ among Itm2a-ZsGreen$^+$cells in the periosteum (left), the growth plate (middle) and bone marrow (right). Mice were analyzed 2 days after the tamoxifen treatment at 4 weeks of age.

(F) The percentage of Itm2a-mCherry$^+$ among Itm2a-ZsGreen$^+$cells calculated from Figure S3C. n = 3 mice per condition from 3 independent experiments.

(G) Experimental design for tamoxifen induction in *Itm2a-CreER; R26-Ai6* mice.

(H) Representative confocal images of staining with the Itm2a antibody, Itm2a-CreER-ZsGreen and Itm2a-mCherry expression patterns in the periosteum. Scale bar: 100 μm.

Fig. S4 *Itm2a-CreER*-labelled cells can self-renew and display multipotency *in vitro*, related to Figure 3.

(A) Representative confocal imaging of the expression of Ctsk, Gli1, PDGFRα, Sca1, CD51, Postn, Osx, and CD34 in *Itm2a* expressing cells (mCherry$^+$ cells) in femur sections from 4-week-old *Itm2a-CreER; R26-Ai6* mice. n = 3 mice from 3 independent experiments. Scale bar: 50 μm.

(B) Experimental design for tamoxifen induction of *Itm2a-CreER; R26-Ai6* mice.

(C) Representative confocal imaging of the periosteum in femur sections from *Itm2a-CreER; R26-Ai6* mice at P3, 1 M or 3 M after tamoxifen dosing at P1. n=3 mice per condition from 3 independent experiments. Scale bar: 100 μm.

(D) Flow cytometry analysis of the percentage of ZsGreen$^+$ periosteal cells from *Itm2a-CreER; R26-Ai6* mice at P3, 1 M or 3 M after tamoxifen dosing at P1. n = 3 mice per condition from 3 independent experiments. Data are presented as the means ± sd.

(E) Representative confocal images of *Itm2a-mCherry* expression patterns in primary cultured periosteum cells with Ki67 immunostaining. And staining with the Bodipy, Sox9 and Runx2 antibodies and *Itm2a-mCherry* expression patterns single cell level.

Fig. S5 Itm2a$^+$ P-SSCs generate cells in the external callus rather than bone marrow upon injury, related to Figure 4.

(A) Representative confocal images of *Itm2a* lineage cells (ZsGreen$^+$ cells) colocalized with an osteoblastic marker (OPN) at the fracture sites from *Itm2a-CreER; R26-Ai6* mice at 14 dpf. n=3.

(B) Representative confocal images of bone drill hole sites in sections from *Itm2a-CreER; Ai6* mice at 7 dpf after tamoxifen dosing. n=4 mice per condition from 4 independent experiments.

(C) Representative confocal images of *Itm2a* lineage cells (ZsGreen$^+$ cells) colocalized with a chondrogenic marker (COL2A1) at the injury sites from *Itm2a-CreER; R26-Ai6* mice in the bone scratch model at 7 dpf. n=3 mice per condition from 4 independent experiments.

(D) The percentage of *Itm2a* lineage cells (ZsGreen$^+$ cells) among COL2A1$^+$ cells calculated from Figure S5C. n=3 mice per group.

(E) Representative confocal images of *Itm2a* lineage cells (ZsGreen$^+$ cells) colocalized with an osteoblastic marker (OPN) at the injury sites from *Itm2a-CreER; R26-Ai6* mice in bone drill and bone scratch model at 14 dpf. n=4 mice per condition from 4 independent experiments.

(F) The percentage of *Itm2a* lineage cells (ZsGreen$^+$ cells) among OPN$^+$ cells calculated from Figure S5E. n=4 mice per group.

Fig. S6 *Prrx1-CreER*-labelled cells contribute to bone fracture healing, related to Figure 4.

(A) Representative confocal images of *Prrx1* lineage cells (ZsGreen$^+$ cells) distribution in periosteum (PO) and bone marrow (BM) regions at 2 days after tamoxifen injection in 4 weeks old *Prrx1-CreER; R26-Ai6* mice. Scale bar: 100 μm.

(B) Experimental design for tamoxifen induction, bone fracture and tissue analysis in *Prrx1-CreER; R26-Ai6* mice.

(C) Representative confocal images of *Prrx1* lineage cells (ZsGreen$^+$ cells) colocalized with an osteoblastic marker (OPN) at the bone fracture sites from *Prrx1-CreER; R26-Ai6* mice at 14 dpf. n=4 mice per condition from 4 independent experiments.

(D) The percentage of *Prrx1* lineage cells (ZsGreen$^+$ cells) among OPN$^+$ cells calculated from Figure S6C. n=4 mice per group.

Fig. S7 Lineage tracing for *Prrx1-CreER; R26-LSL-Ai6; Itm2a-DreER; R26-RSR-tdTomato* mice, related to Figure 5.

(A) Experimental design for tamoxifen induction, bone fracture model, and tissue analysis. dpf, days post fracture.

(B&C) Representative confocal imaging of *Itm2a* lineage cells (tdTomato$^+$ cells) and *Prrx1* lineage cells (ZsGreen$^+$ cells) in femur (B) and vertebra (C) sections from *Prrx1-CreER; R26-LSL-Ai6; Itm2a-DreER; R26-RSR-tdTomato* mice that had been tamoxifen treated at P1. Mice were analyzed 8 weeks after the treatment. n = 3 mice per condition from 3 independent experiments. Scale bar: 100 μm.

(D) Representative confocal images of *Itm2a* lineage cells (tdTomato$^+$ cells) and *Prrx1* lineage cells (ZsGreen$^+$ cells) colocalized with chondrocytes (COL2A1) at the fractured femoral from *Prrx1-CreER; R26-LSL-Ai6; Itm2a-DreER; R26-RSR-tdTomato* mice at 7dpf. Scale bar: 100 μm.

(E) Representative confocal images of *Itm2a* lineage cells (tdTomato$^+$ cells) and *Prrx1* lineage cells (ZsGreen$^+$ cells) colocalized with osteoblasts (OSX$^+$) at the fractured femoral from *Prrx1-CreER; R26-LSL-Ai6; Itm2a-DreER; R26-RSR-tdTomato* mice at 14dpf. Scale bar: 100 μm.

(F&G) The percentage of three cell populations among COL2A1$^+$ chondrocytes, OSX$^+$ osteoblasts calculated from Figure S7E and S7F (Respectively). n = 3/4 mice per condition. Data are presented as the means ± sd.

(H) Representative confocal images of *Itm2a* lineage cells (tdTomato$^+$ cells) and *Prrx1* lineage cells (ZsGreen$^+$ cells) colocalized with osteoblasts (OPN$^+$) at the bone drill-hole model from *Prrx1-CreER; R26-LSL-Ai6; Itm2a-DreER; R26-RSR-tdTomato* mice at 7 dpf. Scale bar: 100 μm.

Fig. S8 Itm2a$^+$ P-SSCs decrease in aged mice.

(A) Experimental design for tamoxifen induction and tissue analysis.

(B) SOFG staining analysis of fractured femurs in 12-week-old mice and 18-month-old mice at 14 dpf. n = 3 mice per condition. Scale bar: 100 μm.

(C) Representative confocal imaging of *Itm2a* lineage cells (ZsGreen$^+$ cells) in 12-week-old mice and 18-month-old mice femur sections from *Itm2a-CreER; R26-Ai6* mice. Mice were analyzed 1 weeks after the treatment. n = 3 mice per condition. Scale bar: 100 μm.

(D) The quantification of the number of *Itm2a* lineage cells (ZsGreen$^+$ cells) in periosteum calculated from Figure S8C. n = 3 mice per condition. Data are presented as the means ± sd.

(E) Representative confocal images of *Itm2a* lineage cells (ZsGreen$^+$ cells) colocalized with osteoblast (OPN) at the fractured femoral from 12-week-old and 18-month-old *Itm2a-CreER; R26-Ai6* mice at 14dpf. Scale bar: 100 μm.

(F) The percentage of *Itm2a* lineage cells (ZsGreen$^+$ cells) among OPN$^+$ osteoblasts calculated from Figure S8E. n = 3 mice per condition. Data are presented as the means ± sd.

Figure S1

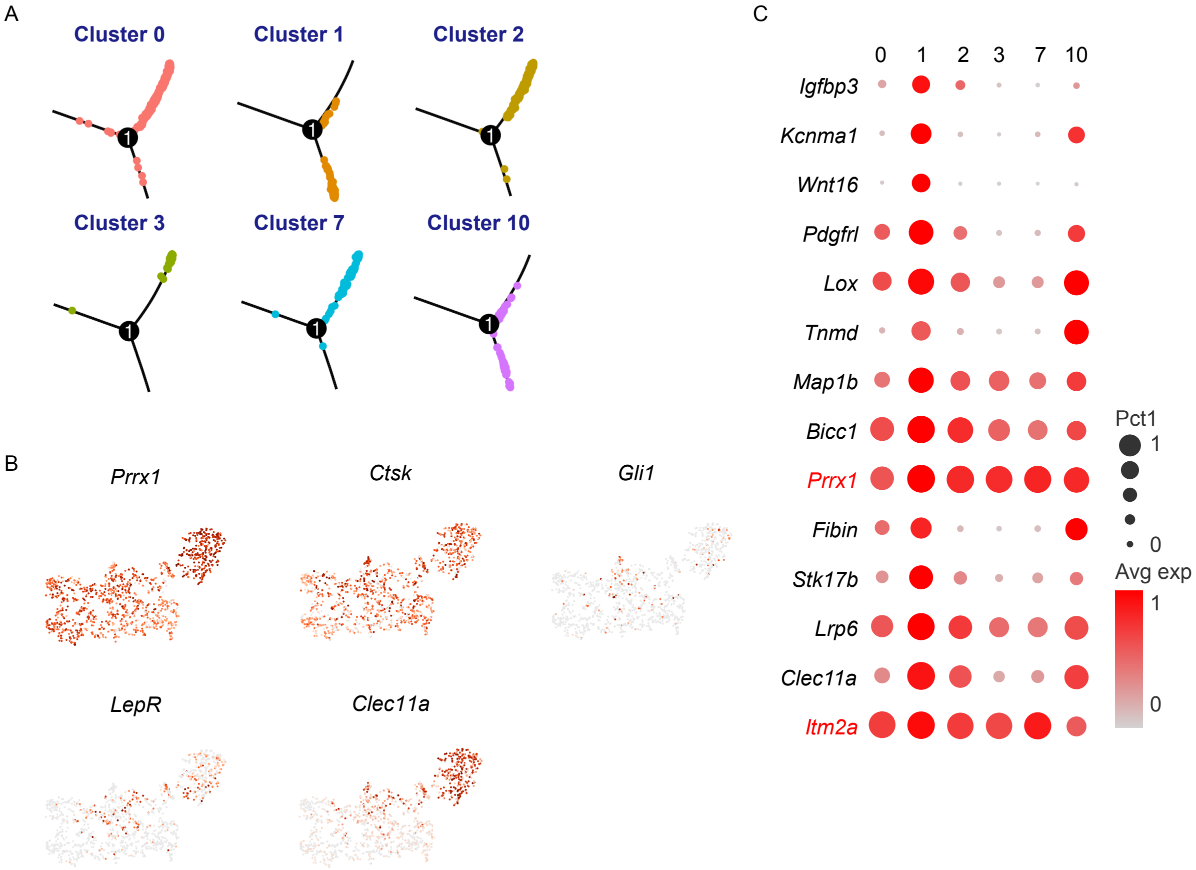
A
Cluster 0
Cluster 1
Cluster 2
Cluster 3
Cluster 7
Cluster 10
B
Prrx1
Ctsk
Gli1
LepR
Clec11a
C
0 1 2 3 7 10
Igfbp3
Kcnma1
Wnt16
Pdgfrl
Lox
Tnmd
Map1b
Bicc1
Prrx1
Fibin
Stk17b
Lrp6
Clec11a
Itm2a
Pct1
1
0
Avg exp
1
0

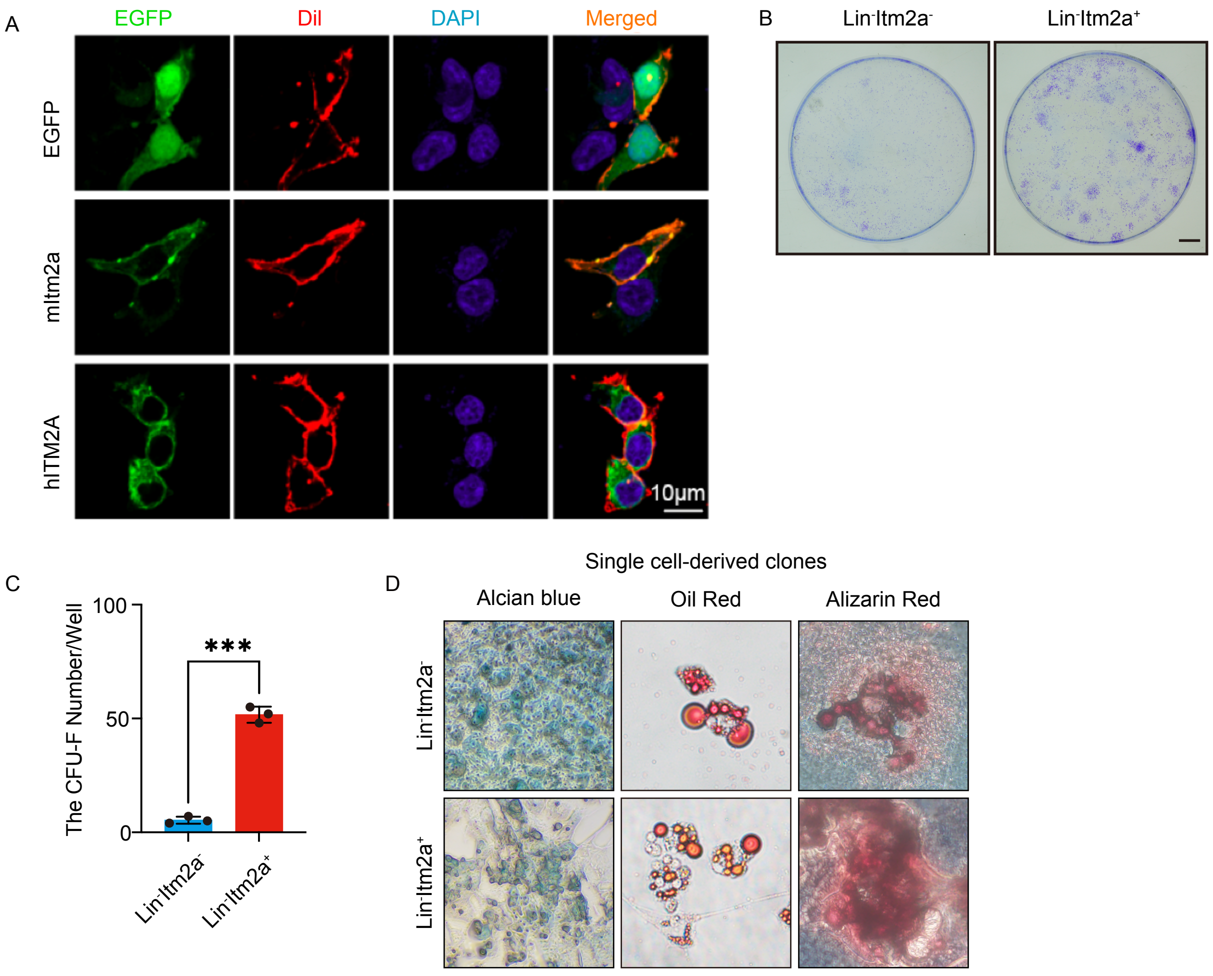
A
EGFP
Dil
DAPI
Merged
EGFP
mItm2a
hITM2A
10μm
B
Lin-Itm2a-
Lin-Itm2a+
C
The CFU-F Number/Well
100
50
0
***
Lin-Itm2a-
Lin-Itm2a+
Single cell-derived clones
D
Alcian blue
Oil Red
Alizarin Red
Lin-Itm2a-
Lin-Itm2a+

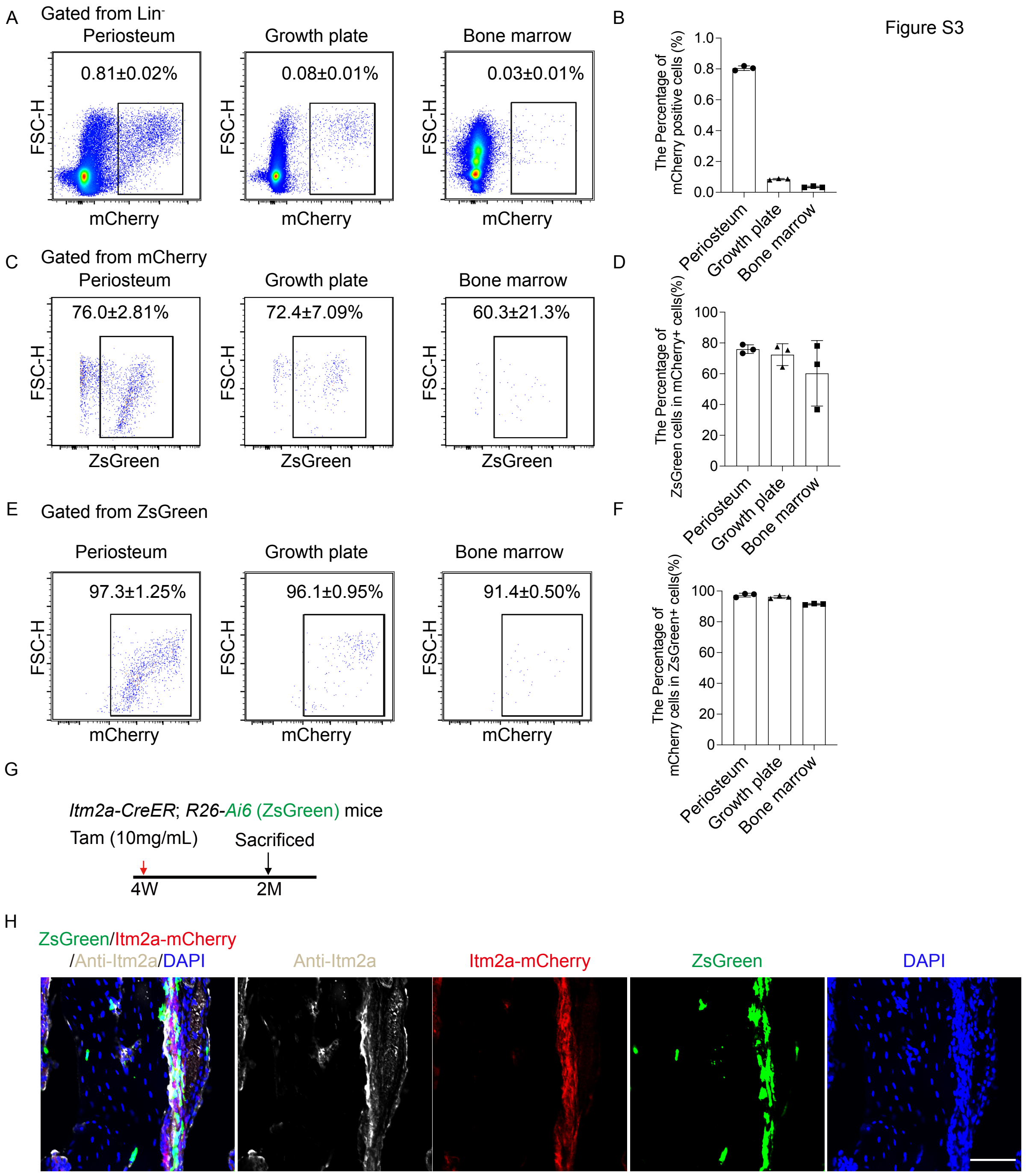
Figure S3
A
Gated from Lin-
Periosteum
Growth plate
Bone marrow
0.81±0.02%
0.08±0.01%
0.03±0.01%
FSC-H
mCherry
B
The Percentage of mCherry positive cells (%)
C
Gated from mCherry
76.0±2.81%
72.4±7.09%
60.3±21.3%
ZsGreen
D
The Percentage of ZsGreen cells in mCherry+ cells(%)
E
Gated from ZsGreen
97.3±1.25%
96.1±0.95%
91.4±0.50%
F
The Percentage of mCherry cells in ZsGreen+ cells(%)
G
Itm2a-CreER; R26-Ai6 (ZsGreen) mice
Tam (10mg/mL)
Sacrificed
4W
2M
H
ZsGreen/Itm2a-mCherry/Anti-Itm2a/DAPI
Anti-Itm2a
Itm2a-mCherry
ZsGreen
DAPI

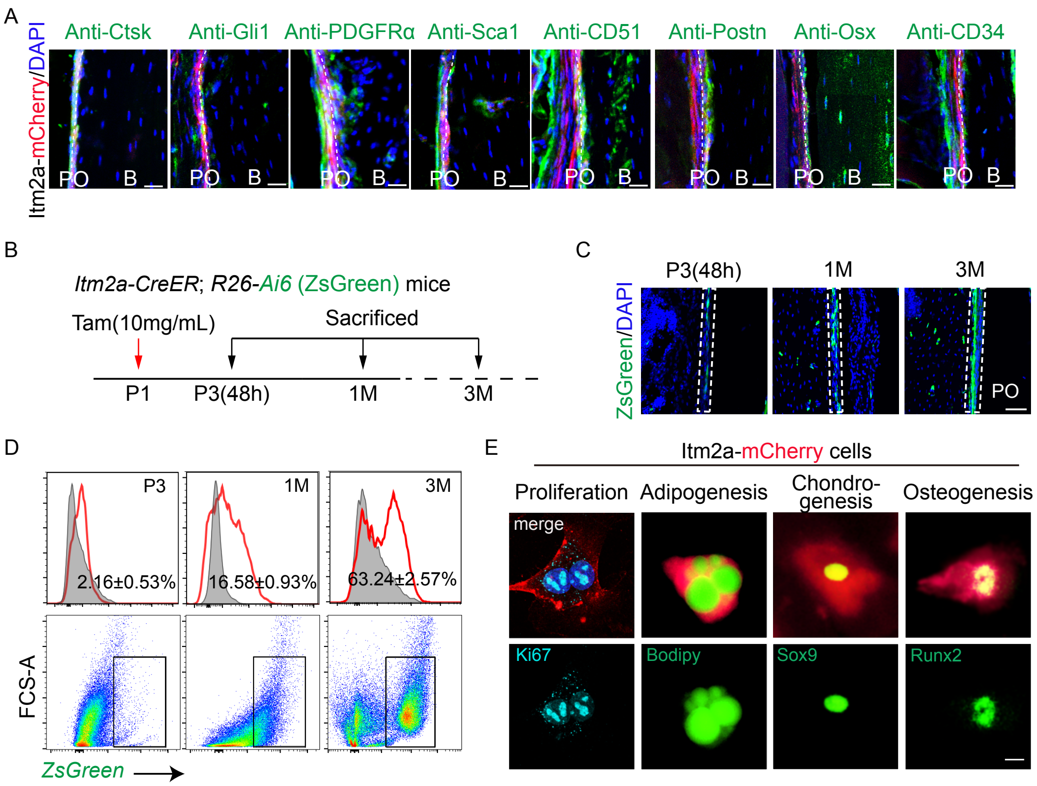
A
Anti-Ctsk
Anti-Gli1
Anti-PDGFRα
Anti-Sca1
Anti-CD51
Anti-Postn
Anti-Osx
Anti-CD34
Itm2a-mCherry/DAPI
PO
B
B
Itm2a-CreER; R26-Ai6 (ZsGreen) mice
Tam(10mg/mL)
Sacrificed
P1
P3(48h)
1M
3M
C
P3(48h)
1M
3M
ZsGreen/DAPI
PO
D
P3
1M
3M
2.16±0.53%
16.58±0.93%
63.24±2.57%
FCS-A
ZsGreen
E
Itm2a-mCherry cells
Proliferation
Adipogenesis
Chondro-genesis
Osteogenesis
merge
Ki67
Bodipy
Sox9
Runx2

Figure S5

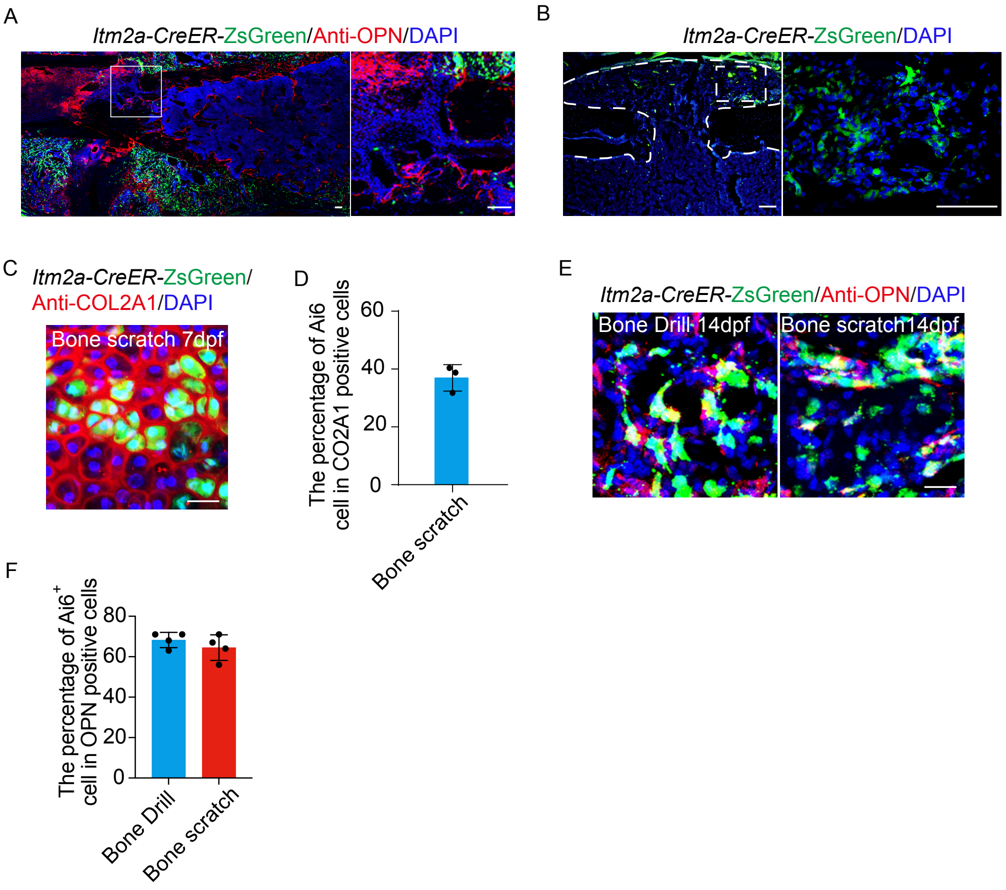
A
Itm2a-CreER-ZsGreen/Anti-OPN/DAPI
B
Itm2a-CreER-ZsGreen/DAPI
C
Itm2a-CreER-ZsGreen/
Anti-COL2A1/DAPI
Bone scratch 7dpf
D
The percentage of Ai6
cell in CO2A1 positive cells
60
40
20
0
Bone scratch
E
Itm2a-CreER-ZsGreen/Anti-OPN/DAPI
Bone Drill 14dpf
Bone scratch14dpf
F
The percentage of Ai6+
cell in OPN positive cells
80
60
40
20
0
Bone Drill
Bone scratch

Figure S6

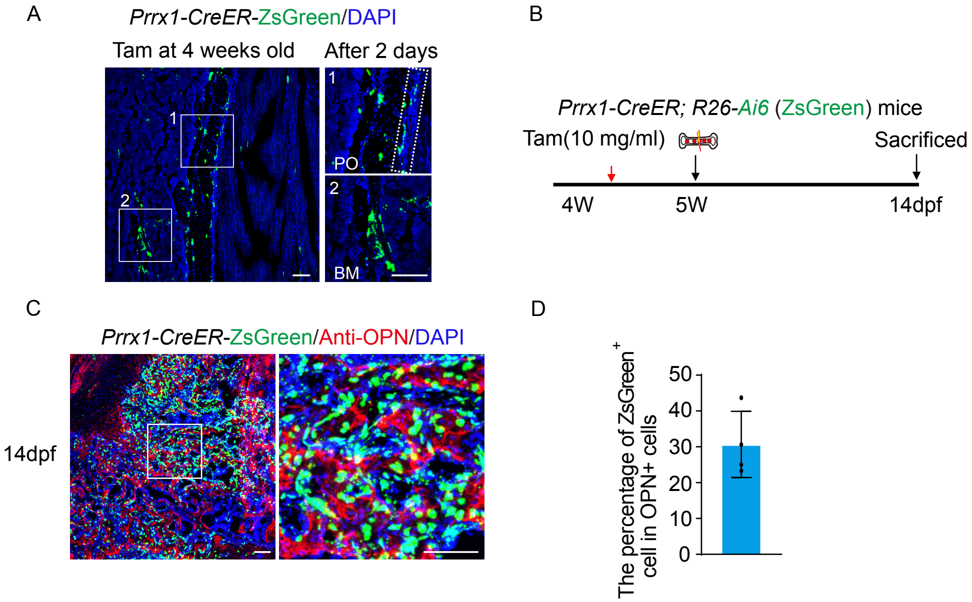
A
Prrx1-CreER-ZsGreen/DAPI
Tam at 4 weeks old
After 2 days
1
2
PO
BM
B
Prrx1-CreER; R26-Ai6 (ZsGreen) mice
Tam(10 mg/ml)
Sacrificed
4W
5W
14dpf
C
Prrx1-CreER-ZsGreen/Anti-OPN/DAPI
14dpf
D
The percentage of ZsGreen+ cell in OPN+ cells
50
40
30
20
10
0

Figure S7

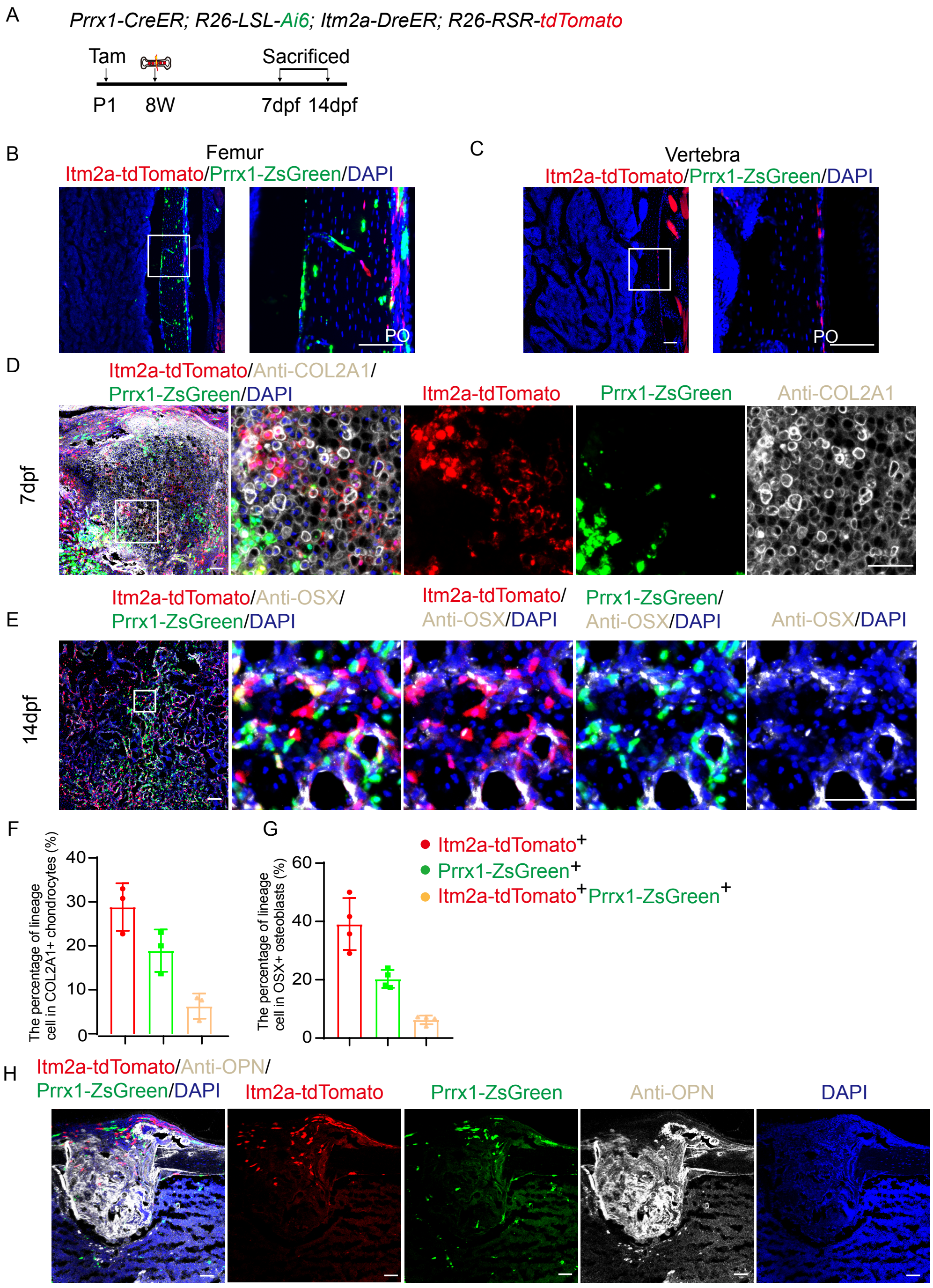
A
Prrx1-CreER; R26-LSL-Ai6; Itm2a-DreER; R26-RSR-tdTomato
Tam
Sacrificed
P1
8W
7dpf
14dpf
B
Femur
Itm2a-tdTomato/Prrx1-ZsGreen/DAPI
PO
C
Vertebra
Itm2a-tdTomato/Prrx1-ZsGreen/DAPI
PO
D
Itm2a-tdTomato/Anti-COL2A1/
Prrx1-ZsGreen/DAPI
Itm2a-tdTomato
Prrx1-ZsGreen
Anti-COL2A1
7dpf
E
Itm2a-tdTomato/Anti-OSX/
Prrx1-ZsGreen/DAPI
Itm2a-tdTomato/
Anti-OSX/DAPI
Prrx1-ZsGreen/
Anti-OSX/DAPI
Anti-OSX/DAPI
14dpf
F
The percentage of lineage
cell in COL2A1+ chondrocytes (%)
40
30
20
10
0
G
The percentage of lineage
cell in OSX+ osteoblasts (%)
60
40
20
0
Itm2a-tdTomato+
Prrx1-ZsGreen+
Itm2a-tdTomato+Prrx1-ZsGreen+
H
Itm2a-tdTomato/Anti-OPN/
Prrx1-ZsGreen/DAPI
Itm2a-tdTomato
Prrx1-ZsGreen
Anti-OPN
DAPI

Figure S8

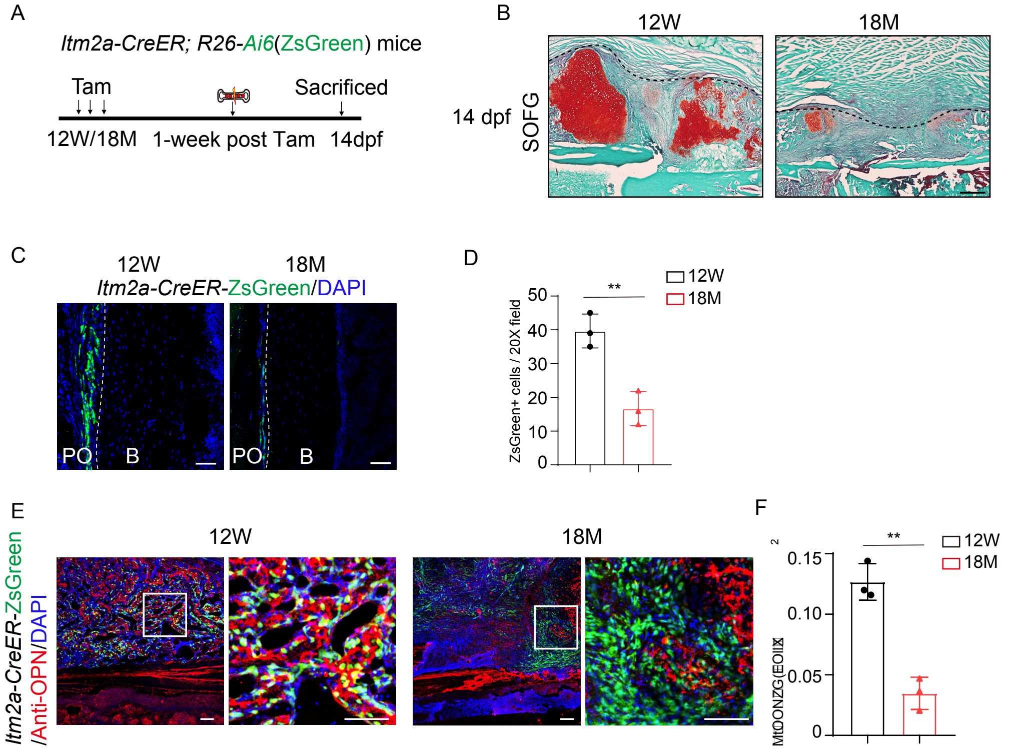
A
Itm2a-CreER; R26-Ai6(ZsGreen) mice
Tam
Sacrificed
12W/18M
1-week post Tam
14dpf
B
12W
18M
14 dpf
SOFG
C
12W
18M
Itm2a-CreER-ZsGreen/DAPI
PO
B
PO
B
D
ZsGreen+ cells / 20X field
50
40
30
20
10
0
**
12W
18M
E
Itm2a-CreER-ZsGreen/Anti-OPN/DAPI
12W
18M
F
0.15
0.10
0.05
0
**
12W
18M
